# Single-Cell RNA Sequencing Analysis of Chicken Anterior Pituitary: A Bird’s-Eye View on Vertebrate Pituitary

**DOI:** 10.3389/fphys.2021.562817

**Published:** 2021-06-29

**Authors:** Jiannan Zhang, Can Lv, Chunheng Mo, Meng Liu, Yiping Wan, Juan Li, Yajun Wang

**Affiliations:** ^1^Key Laboratory of Bio-Resources and Eco-Environment, Ministry of Education, College of Life Sciences, Sichuan University, Chengdu, China; ^2^Key Laboratory of Birth Defects and Related Diseases of Women and Children, Ministry of Education, West China Second University Hospital, Sichuan University, Chengdu, China

**Keywords:** chickens, anterior pituitary, endocrine cell, folliculo-stellate cell, scRNA sequencing, gene expression

## Abstract

It is well-established that anterior pituitary contains multiple endocrine cell populations, and each of them can secrete one/two hormone(s) to regulate vital physiological processes of vertebrates. However, the gene expression profiles of each pituitary cell population remains poorly characterized in most vertebrate groups. Here we analyzed the transcriptome of each cell population in adult chicken anterior pituitaries using single-cell RNA sequencing technology. The results showed that: (1) four out of five known endocrine cell clusters have been identified and designated as the lactotrophs, thyrotrophs, corticotrophs, and gonadotrophs, respectively. Somatotrophs were not analyzed in the current study. Each cell cluster can express at least one known endocrine hormone, and novel marker genes (*e.g., CD24* and *HSPB1* in lactotrophs, *NPBWR2* and *NDRG1* in corticotrophs; *DIO2* and *SOUL* in thyrotrophs, *C5H11ORF96* and *HPGDS* in gonadotrophs) are identified. Interestingly, gonadotrophs were shown to abundantly express five peptide hormones: *FSH, LH, GRP, CART* and *RLN3*; (2) four non-endocrine/secretory cell types, including endothelial cells (expressing *IGFBP7* and *CFD*) and folliculo-stellate cells (FS-cells, expressing *S100A6* and *S100A10*), were identified in chicken anterior pituitaries. Among them, FS-cells can express many growth factors, peptides (*e.g., WNT5A, HBEGF*, Activins, *VEGFC, NPY*, and *BMP4*), and progenitor/stem cell-associated genes (*e.g.*, Notch signaling components, *CDH1*), implying that the FS-cell cluster may act as a paracrine/autocrine signaling center and enrich pituitary progenitor/stem cells; (3) sexually dimorphic expression of many genes were identified in most cell clusters, including gonadotrophs and lactotrophs. Taken together, our data provides a bird’s-eye view on the diverse aspects of anterior pituitaries, including cell composition, heterogeneity, cell-to-cell communication, and gene expression profiles, which facilitates our comprehensive understanding of vertebrate pituitary biology.

## Introduction

It has been well-established that in vertebrates, the anterior pituitary (*Pars distalis*) contains multiple distinct hormone-secreting cell populations, including somatotrophs, lactotrophs, gonadotrophs, corticotrophs, and thyrotrophs. Each cell type is believed to secrete one or two endocrine hormones to regulate many vital physiological processes (Scanes, 2014; Kaiser and Ho, 2016). In mammals, pituitary somatotrophs secrete growth hormone (GH); lactotrophs secrete prolactin (PRL); gonadotrophs produce two gonadotropins, namely follicle-stimulating hormone (FSH) and luteinizing hormone (LH), both of which share a common glycoprotein α-subunit (CGA) and a hormone-specific β-subunit (FSHB and LHB); corticotrophs produce adrenocorticotropin (ACTH), and thyrotrophs secrete TSH, consisting of an α-subunit (CGA, common for FSH and LH) and a hormone-specific β-subunit (TSHB) (Zhu et al., 2007; Edwards and Raetzman, 2018). These pituitary hormones play vital roles in the regulation of growth, development, metabolism, reproduction, and stress (Edwards and Raetzman, 2018). In addition to the endocrine cell population, several non-endocrine/secretory cell populations have also been identified in anterior pituitaries, including progenitor/stem cells, endothelial cells, and folliculo-stellate cells (FS-cells) (Denef, 2008; Gleiberman et al., 2008). Progenitor/stem cells are reported to mainly localize in the periluminal area between anterior lobe and intermediate lobe and sparsely in the parenchyma of mouse anterior pituitaries. These cells can express progenitor/stem-cell markers, *e.g.*, sex-determining region Y-box 2 (SOX2), and have the capacity to differentiate into endocrine cells *in vitro*, thus they are proposed to play critical roles in maintaining organ homeostasis (Fauquier et al., 2008). FS-cells can express S100 proteins specifically and are reported to play important roles in the anterior pituitary, such as supportive effects on endocrine cells, stem cell function, ion transport, phagocytic and catalytic activity (Traverso et al., 1999; Inoue et al., 2002). There is also evidence that FS-cells can secrete growth factors, such as vascular endothelial growth factor (VEGF), fibroblast growth factor 2 (FGF2), leptin (LEP) and Annexin 1 (ANXA1), suggesting a role in paracrine/autocrine communication within the anterior pituitary (Traverso et al., 1999; Denef, 2008).

In mammalian anterior pituitaries, both endocrine cells and FS-cells are likely originated from the oral ectoderm during embryogenesis, although a recent study reported some pituitary endocrine cells are derived from an endodermal origins, at least in fish (Fabian et al., 2020). Under the induction of paracrine signals dorsally from ventral diencephalon (*e.g.*, bone morphogenetic protein 4, BMP4; fibroblast growth factors, FGFs; WNT5A) and ventrally from the oral ectoderm and mesenchyme (*e.g.*, Sonic hedgehog, Shh; bone morphogenetic protein 2, BMP2), the oral ectoderm forms a rudimentary Rathke’s pouch and expresses a specific set of transcription factors spatio-temporally (*e.g., POU1F1, GATA2, NR5A1*, and *TBX19*), which further induces cell differentiation to form distinct endocrine cell lineages (Treier et al., 1998; Zhu et al., 2007; Kelberman et al., 2009; Rizzoti, 2015) in mammals. For instance, in mouse embryonic pituitaries, *POU1F1* (*Pit-1*) expression causes the emergence of three Pit-1 cell lineages (somatotrophs, lactotrophs and thyrotrophs) and *GATA2* and *NR5A1* expression are necessary for specification of the gonadotrophs (Kelberman et al., 2009). After birth, these endocrine cell populations further expand and differentiate under the influence of hypothalamic and peripheral signals, thus forming a functional endocrine gland in vertebrates (Edwards and Raetzman, 2018).

Although pituitary cell populations and functions have been extensively studied in vertebrates, the gene expression profiles of each pituitary cell population remains poorly understood. In recent years, the transcriptome of each pituitary cell population in vertebrate pituitaries by single-cell RNA sequencing (scRNA-Seq) was available for rat (Fletcher et al., 2019), mouse (Cheung et al., 2018; Ho et al., 2020), human (Zhang et al., 2020), zebrafish (Fabian et al., 2020) and medaka (Siddique et al., 2020). However, such study is lacking in other non-mammalian vertebrates including birds. This limitation, undoubtedly, prevents our better understanding of vertebrate pituitary biology. Like mammalian anterior pituitaries, avian anterior pituitaries contain five hormone-secreting cells, including somatotrophs, lactotrophs, thyrotrophs, gonadotrophs, and corticotrophs. These endocrine cell populations secrete GH, PRL, TSH, LH (and FSH), and ACTH, respectively, to regulate avian growth, metabolism, reproduction, and stress (Scanes, 2014). As in mammals, the secretion and/or expression of each pituitary hormone has been reported to be controlled by signals from the hypothalamus and peripheral tissues in birds (Scanes, 2014). GH secretion is controlled by hypothalamic GH-releasing hormone (GHRH), thyrotropin-releasing hormone (TRH) and somatostatin (SST) (Harvey et al., 2014; Meng et al., 2014; Bu et al., 2016). LH secretion is controlled by hypothalamic gonadotropin-releasing hormone 1 (GnRH1). PRL secretion is controlled by hypothalamic vasoactive intestine polypeptide (VIP), TRH, arginine vasotocin (AVT) and negatively, by dopamine (DA) (Bu et al., 2016; Lv et al., 2018). ACTH secretion is controlled by corticotropin-releasing hormone (CRH), AVT and glucocorticoids (CORT), and TSH secretion is controlled by CRH, SST, glucagon-like peptide (GCGL), and thyroid hormones (T4/T3) (Huang et al., 2014; Bu et al., 2019; Wu et al., 2019).

Despite the similarity in pituitary cell populations, functions, and their regulatory mechanisms across vertebrates, we and others have reported functional differences in anterior pituitary between birds and mammals. For instance, chicken anterior pituitaries can produce two novel peptide hormones: gastrin-releasing peptide (GRP) and cocaine-and amphetamine-regulate transcript (CART, also named CARTPT) (Cai et al., 2015; Mo et al., 2017, 2019). The functional conservation and difference of anterior pituitary between mammals and birds promotes us to further investigate the gene expression profiles of each pituitary cell types in birds. Therefore, using chicken as an animal model, our present study aims to: (1) identify the major pituitary cell clusters; (2) investigate the gene expression profiles of each pituitary cell population. Our data, for the first time, revealed the gene expression profiles of each anterior pituitary cell population in an avian model.

## Materials and Methods

### Ethics Statement

Adult chickens (Lohmann layer) used in this study were purchased from local commercial companies. Single cell suspensions were prepared from anterior pituitaries of sexually mature chickens (male: *N* = 6, female: *N* = 6) at 1-year-old stage. All animal experiments were conducted in accordance with the Guidelines for Experimental Animals issued by the Ministry of Science and Technology of People’s Republic of China. All animal experimental protocols were approved by the Animal Ethics Committee of the College of Life Sciences, Sichuan University (Chengdu, China).

### Chemicals, Antibodies, and Primers

All chemicals were purchased from Sigma (Sigma-Aldrich, St. Louis, MO, United States). Rabbit polyclonal anti-NPY antibody (ab10980) was purchased from Abcam (Cambridge, MA, United States) and Donkey anti-rabbit IgG (H + L) cross adsorbed secondary antibody (Dylight 488 conjugate) was purchased from ThermoFisher Scientific (Waltham, MA, United States). All primers were synthesized by Beijing Genome Institute (BGI, Shenzhen, China) and listed in Supplementary Table 1.

### Single-Cell Dissociation and 10x Genomics Chromium Library Construction of Adult Chicken Anterior Pituitary

Pituitary cell dispersion was carried out as described in our previous studies (Meng et al., 2014). Briefly, anterior pituitaries were separated from adult male and female chickens (1-year-old). Then, six male/female pituitaries were pooled and washed in 1 × Hank’s balanced salt solution (HBSS) thrice. These pituitaries were treated by 0.25% trypsin solution for 30 min at 37°C. Dissociated cells were filtered through a 40-μm cell strainer and subject to centrifugation at 500 *g* for 3 min. Then the cell pellet was re-suspended in a solution containing 0.1% bovine serum albumin (BSA) in 1 × PBS solution (calcium- and magnesium-free). Cell viability and numbers were estimated by trypan blue staining. Then, pituitary cells were loaded onto the Chromium system (10x Genomics 3′ GEX V2) to produce cDNA libraries according to the Chromium Single Cell 3′ Reagents Kits V2 User guide (10x Genomics Inc., Pleasanton, CA, United States). The cDNA libraries were quantified by Agilent Tapestation System (Agilent Technologies, Santa Clara, CA, United States) and sequenced by BGI500 (BGI, Shenzhen, China).

### scRNA-Seq Analysis and Data Visualization

For RNA-seq analysis of single pituitary cells, the raw base call (BCL) files generated from the BGI500 system were demultiplexed into paired-end, gzip-compressed FASTQ files using the Cell Ranger software v1.3.1 provided by 10x Genomics (Supplementary Figure 1). Then, both pairs of FASTQ files were provided as input and converted to gene-count matrix using the alevin software v1.4.0 (Srivastava et al., 2019), which is a fast end-to-end pipeline to process droplet-based single-cell RNA sequencing data, cell barcode detection, read mapping, unique molecular identifier (UMI) deduplication and gene count estimation. Quality control, read alignment (with reference to the GRCg6a transcriptome downloaded from the Ensembl database (Ensembl release 103^1^) and raw count quantification for each cell was achieved using the alevin pipeline with default command line options.

Using tximport packages v1.18.0 (Soneson et al., 2015), the counts matrix and associated metadata processed through Alevin were then imported to Seurat package v4.0 (Butler et al., 2018; Stuart et al., 2019; Hao et al., 2020) in R to clusters and further analyzed, using default parameters unless specified otherwise. We first filtered out genes detected in fewer than five cells and those cells containing more than 2.5% mitochondria reads or less than 200 unique genes detected. Doublets that may affect clustering analysis were detected and eliminated using the R package DoubletFinder v.2.0.3 with default command line options (Assuming 15% doublet formation rate) (McGinnis et al., 2019). After this initial quality control (QC) filtering process, 9, 919 cells from female pituitaries and 6, 733 cells from male pituitaries were used for further analysis. After filtering and normalization, independent pituitary samples from the female and male chickens were integrated by Canonical Correlation Analysis, identifying common sources of variation to align the datasets. The top 2000 highly variable genes were identified and used as input for dimensionality reduction via canonical correlation analysis. Data dimensionality reduction was performed using a principal component analysis (PCA), and the first 30 principal components were used in the downstream analyses.

According to Seurat ‘FindClusters’ function (using 30 PCs given the highly variable genes and a resolution of 0.5), all cells were partitioned into 16 distinct clusters, and visualized using Uniform Manifold Approximation and Projection (UMAP) (Becht et al., 2018). According to Seurat ‘FindAllMarkers’ function (min.pct = 0.25, log2fc.threshold = 0.25, *p*_val_adj < 0.001) using the MAST test (Finak et al., 2015), unique cluster-specific marker genes were identified. The selected marker genes for each cell cluster with high log2fc.threshold are listed in Table 1. Cell types were determined using a combination of marker genes identified from the literature (described in section “Cell Populations Identified in Adult Chicken Anterior Pituitary”). Clusters that appeared to correspond to the same cell types were merged. Significantly upregulated genes (signature genes) between female and male samples were found using a MAST test implemented in Seurat by the ‘FindMarkers’ function (min.pct = 0.25, only.pos = TRUE, log2fc.threshold = 0.5, *p*_val_adj < 0.001). The violin plots, feature plot, and dotplot were generated using Seurat software. Cell communication analysis was based on the network analysis and pattern recognition approaches provided by CellChat (version 1.0.0) R package (Jin et al., 2021). Standard workflow was used to predict major signaling inputs and outputs of cells and how these cells and signals coordinate for functions.

**TABLE 1:** Selected marker genes identified in eight chicken anterior pituitary cell clusters.

Cell cluster	Selected marker genes
Gonadotrophs	*GRP, LHB, RLN3, ENSGALG00000045362, C5H11ORF96, HPGDS, CPLX1, CART, HEXB, NR5A1, FAM189A2, FSHB, GNRHR, NRSN1, NFASC, CGA, RRBP1, GPC4, ZFPM1, ENSGALG00000001805, FHL3, KANK1, ENSGALG00000053991, PDIA3, AHCYL1, ENSGALG00000007147, DPP7, SCD, FDPS, EPN3, TSPAN6, LAPTM4B, TESC, SORL1, FDFT1*
Lactotrophs	*PRL, CD24, ENSGALG00000003644, TNFAIP8, HSPB1, SOCS3, SIX3, H3F3B, POU1F1, PKIB, PLK2, STMN1, TSC22D1, TUBA1A, EGR1, CISH, BHLHE40, ANGPTL4*
Thyrotrophs	*TSHB, CGA, CHGB, DIO2, CHGA, SOUL, CALB2, CCNA1, ENSGALG00000052026, PTN, RGS2, QDPR, FKBP11*
Corticotrophs	*POMC, NPBWR2, CALB2, NDRG1, KRT7, DKK3, C12orf75, RGS2*
FS-cells	*S100A6, S100A10, WNT5A, S100A1, ENSGALG00000011190, S100A11, SNCG, KRT24, ALDH1A3, F3, PEBP4, AKR1B10, CD99, SPARCL1, INHBA, CST3, SPARC, TGFB2, CCDC80, CSRP1, HBEGF, ID3, FAM107B, CDO1, APLP2, NDRG1, VIM, PMP22, IFI27L2, ID2, CTSS, IFI6, RASL11A, GLUL, MT4, ENSGALG00000047664, PLBD1, CLDN1, NPY, SELENBP1, BF1, PITX2, ANXA5, BF2, ENSGALG00000045738, CDK6, MARCKS, CYR61, PITX1*
Endothelial cells	*IGFBP7, CFD, TFPI2, PODXL, APOLD1, RAMP2, ENSGALG00000044406, ITIH5, CD74, ENSGALG00000035994, TCIM, ENSGALG00000023472, SPARC, ENG, KLF2, ENSGALG00000045684, EHD3, ABCB1, EGFL7, KDR, BLB2, STARD3, SGK1, RARRES1, PALD1, TMSB4X, HES4, BLB1, LPIN2, B2M, RGS2, BF1, KARS, H3F3C, HSPB9, TIMP3, CARHSP1, TSC22D4, TPM2, ENSGALG00000045199, ENSGALG00000042023, CD81, ENSGALG00000026970*
White blood cells (WBC)	*ENSGALG00000049450, JCHAIN, CRIP1, TXNDC5, IFI30, IRF4, VIM, LMAN1L, CD74, ENSGALG00000050515, BLB2, FKBP11, GNLY*
Red blood cells (RBC)	*IFI27L2, ENSGALG00000047627, CA2, ENSGALG00000038671, CREG1, ENSGALG00000022875, ENSGALG00000013101, GPX1, AK2, TAL1, C15orf48, WBP4, SLC4A1, ENSGALG00000047208, ENSGALG00000053682, ENSGALG00000051188, TMEM183A, YF5, SYNM, RBM38, ENSGALG00000031149, ENSGALG00000040101, BCL2L1, BF1, HBAD, BAG5, BF2, CYB5A, HBBA, HBA1, FTH1*

*Notes: The full-names of these marker genes and their adjusted *P*-values were given in the Supplementary Data 1.*

### Immunofluorescence Staining

Anterior pituitaries collected from 3-week-old male chicks or adult male chickens were fixed in 4% paraformaldehyde (PFA) at 4°C overnight, embedded in paraffin wax, and then sectioned at a thickness of 8 μm. Deparaffinization and immunofluorescence staining were then performed as described in our recent studies (Meng et al., 2014; Mo et al., 2017). Briefly, pituitary sections were deparaffinized in xylene, and rehydrated. Antigen retrieval was performed by heating in citrate buffer (pH = 6) at 95°C for 10 min. After blocking by 5% BSA in PBS for 1 h at room temperature, the sections were incubated with the primary rabbit polyclonal anti-NPY antibody (1:500) at 4°C overnight. After PBS wash, the sections were incubated with fluorochrome-conjugated secondary antibodies (1:300) for 1 h at room temperature. Finally, the pituitary sections were counterstained with DAPI and observed under a fluorescence microscope (Nikon ECLIPSE Ti, Nikon Instruments Inc., United States). Pituitary sections incubated with pre-immune rabbit serum, instead of anti-NPY antibody, were used as a negative control.

### Quantitative Real-Time RT-PCR (qPCR) and Transcriptomic Data Analysis

Total RNA was extracted from chicken pituitaries using RNAzol (Molecular Research Center, Cincinnati, OH, United States) according to the manufacturer’s instructions and reversely transcribed using M-MLV reverse transcriptase (Takara Bio, Shiga, Japan). In brief, oligodeoxythymide (0.5 μg) and total RNA (2 μg) were mixed in a total volume of 5 μL, incubated at 70°C for 10 min, and cooled at 4°C for 2 min. Then, the first strand buffer, 0.5 mM each deoxynucleotide triphosphate and 100 U Moloney murine leukemia virus (MMLV) reverse transcriptase were added into the reaction mix in a total volume of 10 μL. Reverse transcription (RT) was performed at 42°C for 90 min. RT samples were then used to examine the mRNA expression of target genes in chicken anterior pituitaries by quantitative real-time PCR (qPCR) assay on the CFX96 Real-time PCR Detection System (Bio-Rad Richmond, CA, United States), as described in our previous study (Zhang et al., 2017, 2018).

RNA-Seq data obtained in our laboratory was used to evaluate the mRNA expression level of *RLN3* gene (accession no: MT263682) in granulosa cells of growing ovarian follicles (6 mm follicle, F5 preovulatory follicle, and F1 preovulatory follicles) from egg-laying hens (Zhu et al., 2019). In brief, using Tophat v2.0.12 as a mapping tool (Trapnell et al., 2012), clean reads were filtered from raw reads and mapped to the reference genome of *Gallus gallus*^2^. HTSeq v0.6.1 was used to count the reads mapped to each gene. The FPKM (Fragments per kilobase of transcript per million mapped reads) values were used as an indicator of the relative abundance of *RLN3* at different stages.

### Data Analysis

The relative mRNA levels of selected target genes were first calculated as the ratios to that of β*-actin* and then expressed as the fold difference compared to the male/female anterior pituitaries. The data was analyzed by Student *t*-test (between two groups) using GraphPad Prism 7 (GraphPad Software, San Diego CA, United States).

## Results and Discussion

### Cell Populations Identified in Adult Chicken Anterior Pituitary

To investigate the gene expression profiles of chicken pituitary cell populations, we dispersed the cells of anterior pituitaries from adult female (egg-laying stage) and male chickens, and performed single-cell RNA-seq (scRNA-Seq) analysis using the 10x Genomics system (Macosko et al., 2015). A total of 16, 652 pituitary cells passed the quality control check: 9, 919 cells for the female pituitary pool (six pituitaries), and 6, 733 cells for the male pituitary pool (six pituitaries).

**FIGURE 1:**
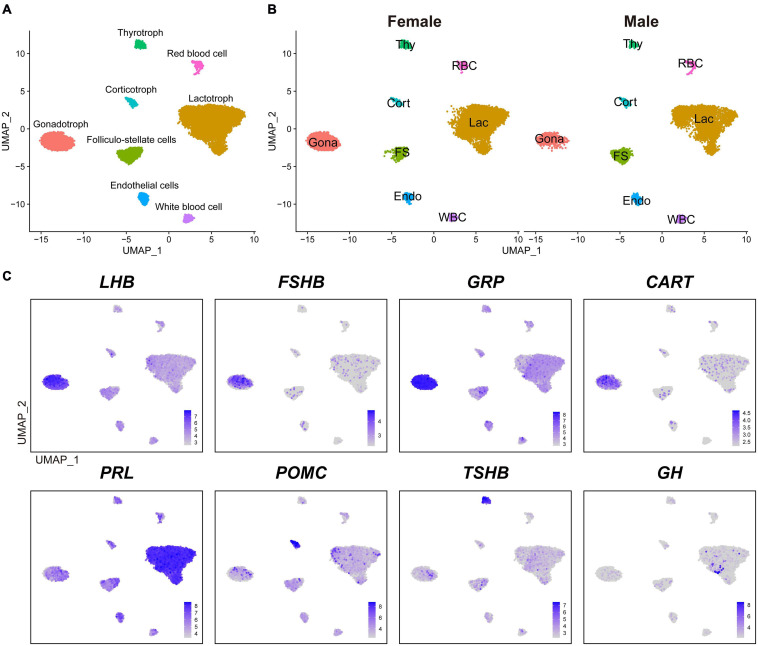
The major cell clusters identified in adult chicken anterior pituitaries. **(A)** Uniform Manifold Approximation and Projection (UMAP) map showing the identified eight pituitary cell types based on the transcriptomes of 16, 652 cells. Cells are colored by Seurat clustering and annotated by cell types (each point represents a single cell). Gona, gonadotrophs; Lac, lactotrophs; Cort, corticotrophs; Thy, thyrotrophs; FS, folliculo-stellate cells; Endo, endothelial cells; WBC, white blood cells; RBC, red blood cells; UMAP, Uniform Manifold Approximation and Projection. **(B)** UMAP maps of the eight cell types identified in female (9, 919 cells) and male (6, 733 cells) adult chicken anterior pituitaries. **(C)** UMAP maps showing the abundant expression of pituitary hormone(s) in distinct cell clusters (*LHB, FSHB, GRP*, and *CART* are abundantly expressed in Gona-cluster, *PRL* in Lac-cluster, *POMC* in Cort-cluster, *TSHB* in Thy-cluster, and *GH*). Color bar indicates natural log transformed normalized expression.

We performed a canonical correlation analysis on gene expression matrix and integrated datasets from male and female pituitaries to classify major cell clusters. As a result, we identified eight major transcriptionally distinct cell clusters. According to the known marker genes of pituitary endocrine cell lineages, we designated four endocrine cell clusters, as lactotrophs (Lac, abundantly expressing *PRL*), gonadotrophs (Gona, abundantly expressing *LHB, FSHB, GRP*, and *CART*), thyrotrophs (Thy, abundantly expressing *TSHB*), and corticotrophs (Cort, abundantly expressing *POMC*) (Figure 1A). These endocrine cell clusters could also be identified in the anterior pituitaries of both sexes, and no sex specific cluster was found (Figure 1B).

In vertebrates, the anterior pituitary contains five classical pituitary endocrine cell populations, including lactotrophs, somatotrophs, gonadotrophs, thyrotrophs, and corticotrophs/melanotropes (Cheung et al., 2018; Fletcher et al., 2019; Ho et al., 2020; Zhang et al., 2020). Except somatotrophs, our scRNA-seq data successfully reveal four major cell clusters in adult chicken anterior pituitaries (Figure 1). Since immunostaining of GH in the chicken pituitary reveal an abundance of somatotroph cells in the caudal lobe (Bu et al., 2016), the lack of somatotroph population is likely due to loss of GH-expressing cells during cell dissociation and collection with sub-optimal cytocentrifugation speed in this study. The low number of GH-positive cells located in lactotrophs did not form a separate cluster during analysis (Figure 1C), which is likely a technical shortcomings of our study and need further investigations.

Excluding somatotrophs, our scRNA-seq data suggest that lactotrophs are likely the largest cell population in adult chicken pituitaries of both sexes (Figure 1B), which is consistent with the observation in adult rodents as revealed by scRNA-seq analyses (Cheung et al., 2018; Fletcher et al., 2019). This result is also consistent with our recent finding that PRL-immunoreactive (PRL-ir) cells are the mainly population in adult female chicken pituitaries (Mo et al., 2017). Previous reports also support the idea that plasma PRL is at an extremely high level in chickens and other birds at various adult reproductive stages (*e.g.*, egg-laying, incubation) (Harvey et al., 1979; Scanes et al., 1979; Sharp et al., 1979). The large proportion of lactotrophs and high plasma PRL levels tend to support the important roles of lactotrophs in the reproduction of both male and female chickens, and possibly in other birds as well (Sharp et al., 1998; Sreekumar and Sharp, 1998).

Besides the four major endocrine cell clusters mentioned above, four non-endocrine/non-secretory cell clusters, including folliculo-stellate cell (FS), endothelial cell (Endo), white blood cell (WBC), and red blood cell (RBC), were also identified in chicken anterior pituitaries (Figure 1), similar to recent findings in rodent anterior pituitaries (Cheung et al., 2018; Fletcher et al., 2019). FS-cluster is known for the expression of marker genes encoding S100 proteins in mammals (*e.g., S100B, S100A6, S100A1*, and *S100A11*) (Denef, 2008; Fletcher et al., 2019) and chickens (*e.g., S100A6, S100A10, S100A1*, and *S100A11*) (Supplementary Figure 2). Endo-cluster expresses marker genes, such as insulin like growth factor binding protein 7 (*IGFBP7*) and complement factor D (*CFD*). RBC-cluster expresses marker genes, such as hemoglobin beta, subunit A (*HBBA*) and hemoglobin alpha, subunit D (*HBAD*)*;* WBC-cluster expresses marker genes such as joining chain of multimeric IgA and IgM (*JCHAIN*), cysteine rich protein 1 (*CRIP1*) and lysosomal thiol reductase (*IFI30*) (Table 1). These findings, taken together, suggest that the major cell populations of the anterior pituitary from birds and mammals share common markers.

### Marker Genes Identified in Adult Chicken Anterior Pituitary

**FIGURE 2:**
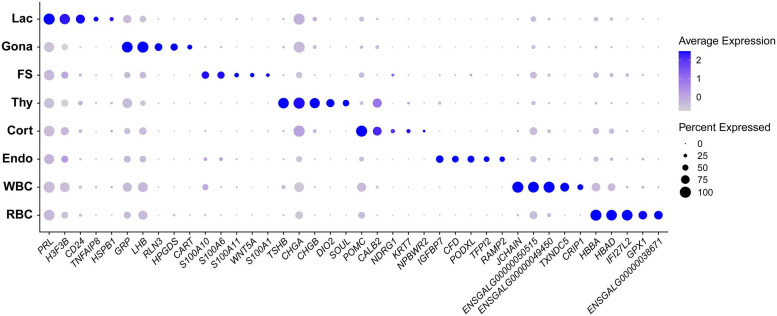
Dot plot showing the expression pattern and level of some established marker genes (shown in columns) for each major cell type (shown in rows) in chicken anterior pituitaries. Color intensities show the expression level of the indicated gene.

**FIGURE 3:**
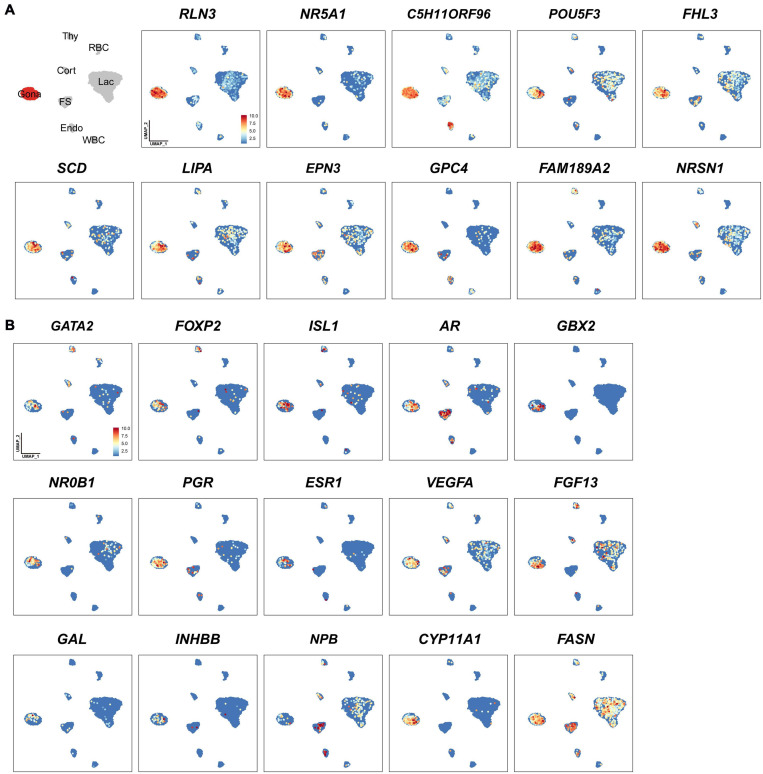
Uniform Manifold Approximation and Projection (UMAP) maps showing the expression of some genes expressed in gonadotrophs of chicken anterior pituitaries. **(A)** The selected marker genes in pituitary gonadotrophs. **(B)** UMAP maps showing the expression of some non-marker genes, including transcription factors (*GATA2, FOXP2, ISL1, AR, GBX2, NR0B1, PGR, ESR1*), growth factor/peptide (*VEGFA, FGF13, GAL*, activin B (encoded by *INHBB*), and *NPB*), and enzymes (*CYP11A1, FASN*) in pituitary gonadotrophs. Color bar indicates the relative expression level of each marker gene from the lowest (light blue dots) to the highest expression level (dark red dots).

In this study, we identified 33-138 marker genes for each pituitary cell cluster (Table 1, Supplementary Data 1) (*Note:* Table 1 shows the selected marker genes for each cell cluster, while all marker genes and their full names for eight cell clusters are listed in Supplementary Data 1). The expression profiles of selected marker genes for each cluster are presented as a dot plot in Figure 2. In the Gona-cluster, we identified 93 marker genes, such as relaxin 3 (*RLN3*), *C5H11ORF96*, hematopoietic prostaglandin-D synthase (*HPGDS*, catalyzing the conversion of prostaglandin H2 to prostaglandin D2), complexin 1 (*CPLX1*), GnRH receptor (*GnRHR*, also named *GnRHR2* in birds), amyloid beta precursor protein (*APP*), and novel transcripts (e.g., ENSGALG00000045362, ENSGALG00000001805, and ENSGALG00000053991) (Table 1 and Figures 2, 3A).

**FIGURE 4:**
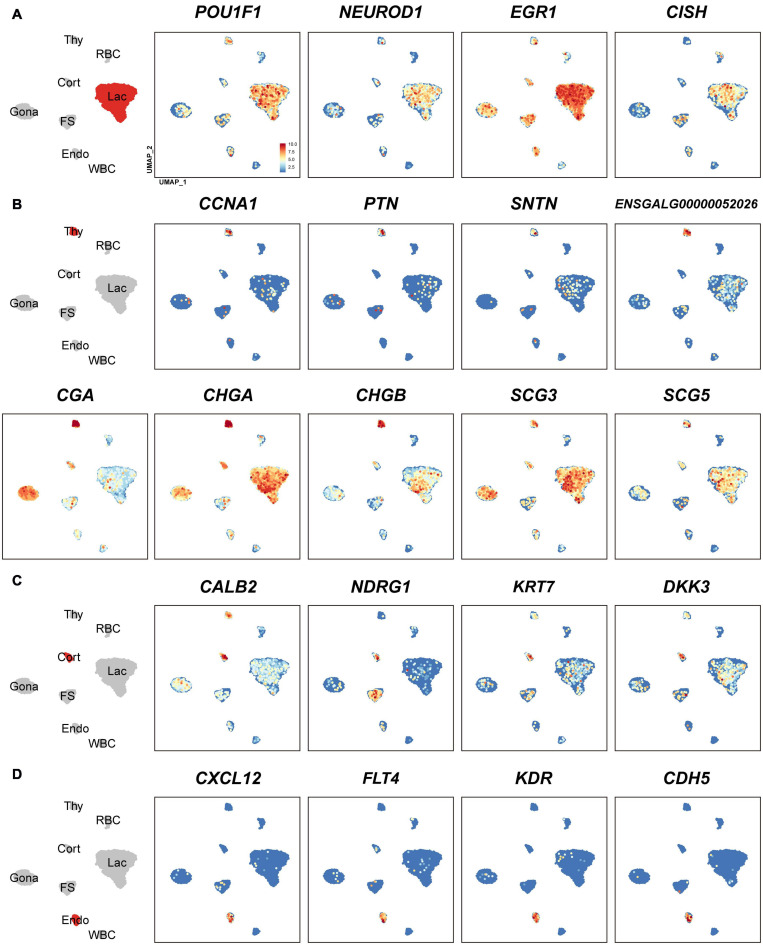
Uniform Manifold Approximation and Projection (UMAP) maps showing the expression of some marker genes in lactotrophs (**A**: Lac), thyrotrophs (**B**: Thy), corticotrophs (**C**: Cort), and endothelial cells (**D**: Endo). Color bar indicates the relative expression level of each marker gene from the lowest expression (light blue dots) to highest expression (dark red dots).

In lactotroph cluster, we identified 138 marker genes, such as *H3F3B, CD24, TNFAIP8, HSPB1, SIX3, POU1F1, NEUROD1, EGR1*, and *ANGPTL4* (Table 1 and Figures 2, 4A). In thyrotroph cluster, 69 marker genes, such as *CGA*, chromogranin A (*CHGA*), chromogranin B (*CHGB*), iodothyronine deiodinase 2 (*DIO2*, an enzyme for conversion of T4 to T3), *SOUL, CALB2, RGS2*, and ENSGALG000000052026 were identified (Table 1 and Figures 2, 4B). In corticotroph cluster, 33 marker genes, such as *CALB2, NDRG1, NPBWR2, KRT7, C12ORF75*, and dickkopf 3 (*DKK3*, an WNT signaling pathway inhibitor) were identified (Table 1 and Figures 2, 4C).

In FS-cell cluster, 74 marker genes were identified, and some of them including *S100A1, S100A6, S100A10, S100A11*, and *ANXA5*, have been reported in fish, birds, and mammals (Table 1 and Figure 2, Supplementary Figure 2) (Van Nassauw et al., 1987; Denef, 2008; Anbalagan et al., 2018; Fletcher et al., 2019). In endothelial cell population, we identified 83 marker genes, such as *RAMP2, CDH5, CXCL12*, VEGF receptors (*FLT4, KDR*), *GPR1, RARRES2, ACVRL1*, and *NOTCH1* (Table 1 and Figures 2, 4D). In blood cells, 137 marker genes were also identified in WBC- and RBC-clusters and listed in the Table 1 and Supplementary Data 1.

Genes like *LHB, FSHB, GnRHR* and transcriptional factors (*e.g., NR5A1*) have been reported in rodent/avian pituitaries (Cheung et al., 2018; Fletcher et al., 2019), and could be identified in the Gona-cluster of both chickens and mammals, indicating their conserved roles in gonadotroph, such as lineage specification and gonadotropin expression/secretion (Edwards and Raetzman, 2018). Likewise, marker genes for lactotrophs (*e.g., EGR1*), thyrotrophs (*e.g., TSHB, CGA, CHGA, CHGB*, and *DIO2*), corticotrophs (*POMC*), FS-cells (S100 proteins and *ANXAs*) and endothelial cells have been identified in chickens or mammals (Fletcher et al., 2019). Further studies on these genes will help to reveal their specific roles in chicken anterior pituitaries.

Through the transcriptomic analysis of single pituitary cell, we identified many non-marker genes of interest in each cell type. As an example, we found that additional transcriptional factors, such as *GATA2, AR, FOXP2* (French et al., 2007), *NR0B1* (also called *DAX-1*, critical for the development and function of adrenal and hypothalamus-pituitary-gonadal axis) (Ikeda et al., 1996; Achermann et al., 2001), *GBX2* (a homeobox gene involved in the hindbrain development during gastrulation) (Waters and Lewandoski, 2006) and *ISL1* (crucial for pancreatic cell lineage development) (Larsson, 1998), are co-expressed in the Gona-cluster (Figure 3B). The question whether all these genes are involved in the control of gonadotroph cell differentiation and functions would be an interesting topic worthy of further investigations. Similarly, several non-marker genes were identified in other cell types (Figure 3B), therefore, future extensive studies are required to address their functions in these pituitary cell populations.

### Endocrine Hormone Genes Are Expressed by Multiple Pituitary Cell Types

Although *PRL* is abundantly expressed in lactotroph cluster, we found that it is also weakly expressed in nearly all other endocrine cell clusters identified. Similarly, *LHB, GRP*, and *CART* highly expressed in gonadotrophs are weakly expressed in the other pituitary cell clusters; *TSHB* and *POMC* mRNAs are abundantly expressed in thyrotrophs and corticotrophs, respectively, and weakly expressed in some other cell clusters (Figure 1 and Supplementary Figure 3). *CGA* encoding α-subunit common for TSH, FSH and LH is abundantly expressed in thyrotroph and gonadotroph clusters and weakly expressed in other cell types (Figure 4B). Like endocrine hormone genes, other genes encoding potential bioactive peptides, *e.g., CHGA, CHGB, SCG3*, and *SCG5* are expressed abundantly in thyrotrophs and moderately in other endocrine cell clusters (Figure 4B).

It is generally accepted that each endocrine cell population can express one or two hormones in the anterior pituitary of vertebrates (Zhu et al., 2007; Kelberman et al., 2009; Scanes, 2014). Recent scRNA research in Japanese medaka confirm a ‘one cell type, one hormone’ division of labor, in which major hormones are produced by a single, dedicated cell type (Siddique et al., 2020). This is different from another scRNA report on the rat pituitary, in which cells are often responsible for producing and releasing multiple hormones (Ho et al., 2020). At least for gonadotrophs, the situation is quite different between fish and other tetrapods. In all fish, LH and FSH are produced by different cells, whereas in most tetrapods they seem to be produced in the same cell (Siddique et al., 2020). There are lines of evidence also showing that one pituitary cell type expressing multiple hormone mRNAs exists in normal rodent pituitaries (Childs et al., 2000; Seuntjens et al., 2002; Villalobos et al., 2004; Childs et al., 2020). In chicken pituitaries, we found that lactotrophs predominantly express *PRL*, however, they can also weakly express *LHB, GRP, GH, POMC*, and *TSHB*. Similarly, gonadotrophs could also weakly express *PRL, TSHB, GH*, and *POMC* (Figure 1 and Supplementary Figure 3). Presumably, the weak expression of multiple hormone genes in one endocrine cell type may be correlated with the weak expression of multiple transcription factors in that population, *e.g., POU1F1, NR5A1* (*SF1*), *GATA2*, and *EGR1* (Supplementary Figure 4), which have been proposed, either alone or in combination, to be expressed in one cell lineage specifically, and thus control its hormone expression (Edwards and Raetzman, 2018). For instance, *POU1F1*, which has been proposed to be exclusively expressed in three Pit-1 lineages (lactotrophs, thyrotrophs, and somatotrophs) and responsible for their differentiation and hormone expression (Edwards and Raetzman, 2018), is also weakly expressed in other cell lineages, including gonadotrophs and corticotrophs (Figure 4A). Future study on the protein expression and secretion of multiple hormones from these cells will help to define the nature of these so-called ‘multi-hormone’ expressing cells (Ho et al., 2020).

### Gonadotrophs Produce Five Hormones: LH, FSH, GRP, CART, and RLN3 in Chickens

**FIGURE 5:**
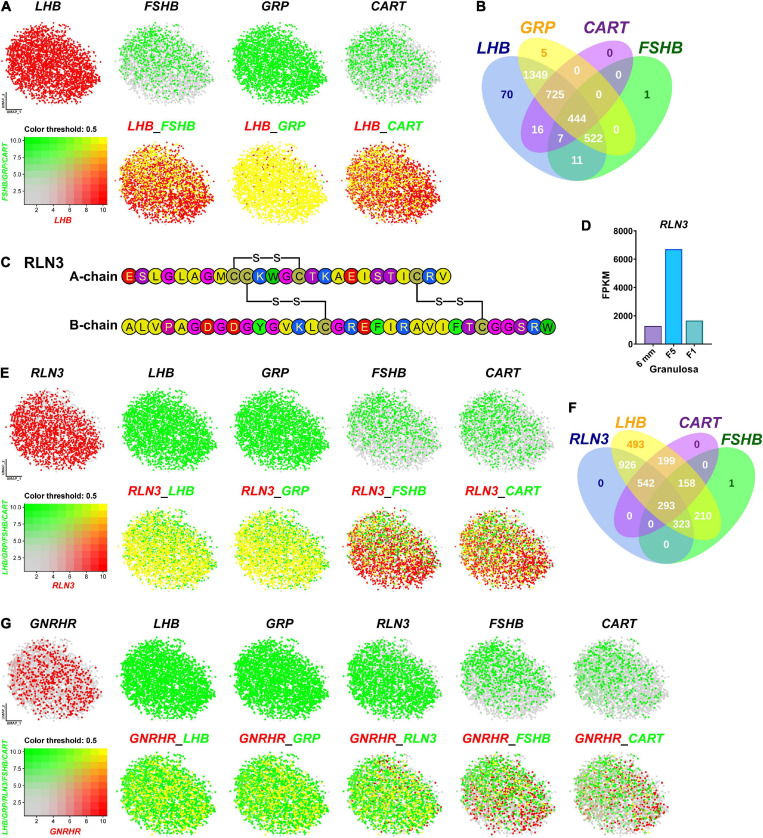
Visualization of the genes co-expressed in gonadotrophs. **(A)** Co-expression of *LHB* and other hormone gene (*FSHB*/*GRP*/*CART*) in gonadotrophs. Gene expression levels are color coded. Red/Green indicates the high expression level and gray indicates the low expression level, as shown in the bottom left corner. Cells abundantly co-expressing *LHB* and *FSHB*/*GRP*/*CART* are shown in yellow. **(B)** Venn diagram showing the numbers of *LHB, GRP, CART* and *FSHB*-positive cells in Gona-cluster. **(C)** Amino acid sequence of the A- and B-chains of chicken *RLN3* (accession no.: MT263682). The three disulfide bonds formed by six cysteine residues (red) are highlighted by red lines. **(D)** RNA-Seq analysis showing the abundant expression of *RLN3* in granulosa cells of growing follicles (6 mm, F5, F1) in adult chicken ovaries. The expression levels of the *RLN3* transcripts were expressed in FPKM values. **(E)** Co-expression of *RLN3* with *LHB*/*GRP*/*FSHB*/*CART* in gonadotrophs. **(F)** Venn diagram showing the numbers of *RLN3-, LHB-, CART-*, and *FSHB*-positive cells in the gonadotroph cluster. **(G)** Co-expression of *GNRHR* (also called *GnRHR2* in birds) and *LHB*/*GRP*/*RLN3*/*FSHB*/*CART* in gonadotrophs.

Since we and others reported that *LHB, GRP, CART*, and *FSHB* are abundantly expressed in chicken anterior pituitaries (Proudman et al., 1999; Mo et al., 2017; Mo et al., 2019), we visualized the co-expression of the four genes using Seurat software. As shown in Figures 5A,B, *FSHB, GRP*, and *CART* are co-expressed in gonadotrophs expressing *LHB* (> 99% overlapping signals). It has long been believed that in birds, two gonadotropins, FSH and LH, are mainly expressed in separate sub-populations of gonadotrophs detected by immuno-histochemistry (Proudman et al., 1999; Puebla-Osorio et al., 2002). However, we found that *FSHB* mRNA is localized in gonadotrophs expressing *LHB* nearly exclusively. Our study clearly indicates that, as in mammals (Gregory et al., 2005) and frog (Pinelli et al., 1996), FSH and LH can be co-expressed in the same gonadotroph cells in chickens (Figure 5A). This ambiguity in chicken may be caused by the characteristics of the immunological reagents used in previous studies, which need further elucidation.

Like *FSHB, GRP* and *CART* are abundantly expressed in gonadotrophs expressing *LHB*. This is also supported by our recent findings in chickens, in which GRP-ir mainly co-localizes with LH-ir cells, and CART peptide secretion is strongly induced by chicken GnRH (Mo et al., 2017, 2019). Our finding on the co-expression of these hormones in gonadotrophs, for the first time, provides convincing evidence on our hypothesis that gonadotrophs can produce the four peptide hormones, *i.e.*, LH, FSH, GRP and CART, in chickens and all of them may be actively involved in the regulation of male/female chicken reproduction (and possibly other physiological processes) (Mo et al., 2019). Like our findings in chickens, CART peptide is reported to be abundantly expressed in rat gonadotrophs (Kuriyama et al., 2004). These findings suggest that in addition to LH and FSH, other peptide hormone (s) (*e.g.*, CART) can be produced by gonadotrophs in avian/mammalian species (Kappeler et al., 2006; Mo et al., 2019).

Besides the four hormones mentioned above, Relaxin 3 (*RLN3*) is abundantly and specifically expressed in the gonadotroph of female (and not male) chickens. RLN3 is a hetero-dimeric peptide (consisting of A-chain and B-chain) and shares structural similarity to insulin (INS) in vertebrates (Halls et al., 2015). In this study, we found that chicken *RLN3* (accession no: MT263682) is a novel marker abundantly expressed in Gona-cluster (Figure 3), which is consisting of A-chain (25 a.a.) and B-chain (33 a.a.) and has three pairs of cysteines involved in disulfide bridges (Figure 5C). Our transcriptomic data demonstrated the extremely abundant expression of *RLN3* in granulosa cells of growing follicles (6 mm, F5, and F1) in adult chicken ovaries (Figure 5D; Zhu et al., 2019). We further visualized its co-expression with *LHB, GRP, FSHB*, and *CART* in Gona-cluster. As shown in Figures 5E,F, nearly all cells expressing *RLN3* overlap with cells expressing *LHB* and *GRP*. In contrast, *RLN3* partially co-localize with *FSHB* and *CART* (∼30%/40%) in gonadotrophs. This finding led us to hypothesize that RLN3 is most likely a ‘novel endocrine hormone’ secreted by pituitary gonadotrophs and ovaries, which may be actively involved in the regulation of female reproduction. In accordance with our hypothesis, pituitary *RLN3* mRNA levels have also been reported to change significantly between brooding and egg-laying chickens and ducks (Shen et al., 2016; Ye et al., 2019). Future studies on RLN3 actions on the anterior pituitary, ovary, oviduct, and other tissues will help to define the actions of this ‘novel hormone’ in birds.

Since GnRHR mediates the effects of GnRH signaling on gonadotroph secretion, we further visualized the co-localization of *GnRHR* with *LHB, GRP, RLN3, FSHB*, or *CART*, respectively. As shown in Figure 5G, *GnRHR*-expressing cells overlap with the majority of *LHB*/*GRP*/*RLN3*-cells, however, *GnRHR* only co-localizes with *FSHB*/*CART* partially. Notably, the expression pattern we reported here is similar to that observed in mature female medaka (Hodne et al., 2019) and in mice during embryogenesis (Wen et al., 2010), where GnRHR is expressed exclusively in LH cells and not in FSH cells. These results suggest that FSH and CART may be more dependent on other regulation factors than GnRH in chicken, which need additional investigations.

### Receptors Expressed in Pituitary Cell Clusters

**FIGURE 6:**
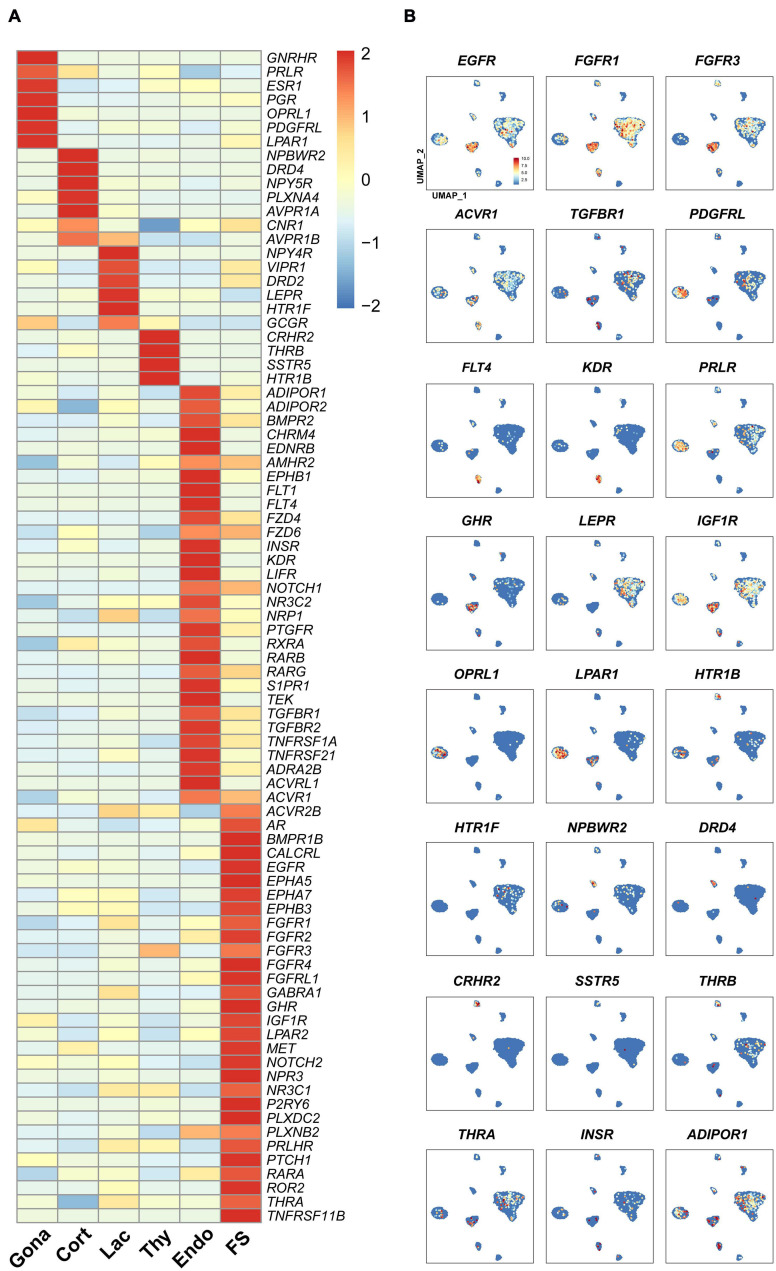
The expression of genes encoding the receptors for endocrine/paracrine/autocrine signals in chicken pituitary cell clusters. **(A)** Heat-map showing the expression of receptor genes in different pituitary cell types. The expression level of each gene was normalized to the z-score and color-coded. **(B)** mRNA signals for some receptor genes shown in **(A)** were projected on the UMAP plots.

In vertebrates, the receptors found in anterior pituitary can receive signals from the hypothalamus and peripheral tissues, such as the gonads, thyroid, and adrenal gland, thus forming multiple closed-loop feedback axes, such as the hypothalamus-pituitary-gonad (HPG), hypothalamus-pituitary-thyroid (HPT), and hypothalamus-pituitary-adrenal (HPA) axes (Vazquez-Borrego et al., 2018). Since receptors can mediate the actions of hypothalamic hormones, peripheral signals, and local pituitary-derived factors, we examined the expression of receptor genes in each pituitary cell type (Figure 6).

In gonadotrophs, the receptors for GnRH (*GnRHR*), nociceptin (*OPRL1*), gonadal steroids (androgen receptor, *AR*; progesterone receptor, *PGR*; estradiol receptor α, *ESR1*), PRL (*PRLR*), SHH (*PTCH1*), PDGFs (*PDGFRL*), and prostaglandin E2 (*PTGER4*) (Kwok et al., 2012) *etc.*, were found to be highly expressed (Figure 6), suggesting that gonadotroph function is likely controlled by hypothalamic input (*e.g.*, GnRH) and peripherals signals from the gonads and extra-gonadal tissues, such as the adrenal gland, pancreas, gastrointestinal tract, liver and adipose tissue (Scanes, 2014).

In corticotrophs, the receptors for NPW (*NPBWR2*), dopamine (*DRD4*), AVT (*AVPR1A, AVPR1B*), NPY (*NPY5R*), and cannabinoid (*CN1R*) *etc.*, were also found to be highly expressed (Figure 6). In lactotrophs, the receptors for hypothalamic factors [*e.g.*, VIP (*VIPR1*), AVT (*AVPR1B*), dopamine (*DRD2*)], peripheral/local signals [*e.g.*, leptin (*LEPR*), serotonin (*HTR1F*), and pancreatic polypeptide PP (*NPY4R*) (Gao et al., 2017)], were found to be expressed (Figure 6). In thyrotroph, the receptors for CRH (*CRHR2*), TH (*THRB*), SST (*SSTR5*), and serotonin (*HTR1B*) *etc.*, were also found to be highly expressed (Figure 6).

Like endocrine cells, endothelial cells also express many receptor genes (Figure 6), including the receptors for VEGFC (*FLT4*), VEGFA (*KDR* and *FLT1*), BMP, FGFs, TGFBs, RA, NOTCH1 ligands, prostaglandin F (*PTGFR*) (Kwok et al., 2012), chemerin (*GPR1*), as well as endothelin (*EDNRB*) critical for endothelial cell differentiation and functions (Liu et al., 2019), suggesting that these hormones may regulate Endothelial cells functions.

In this study, we found that many receptors are expressed in endocrine/non-endocrine cell clusters (Figure 6). All these findings not only support previous pioneering findings regarding the hormonal regulation of avian pituitary gonadotrophs (*e.g.*, GnRH and gonadal steroids) (Hall et al., 1986; Sharp et al., 1987; Millar, 2005), lactotrophs (VIP, dopamine, CORT, and NPW) (Macnamee et al., 1986; el Halawani et al., 1990; Al Kahtane et al., 2003; Fu and Porter, 2004; Bu et al., 2016; Scanes, 2016; Lv et al., 2018), thyrotroph (CRH, T3, GCGL and SST) (De Groef et al., 2003; De Groef et al., 2007; Huang et al., 2014), and corticotroph functions (AVT, CRH, and CORT) (Mikhailova et al., 2007; Selvam et al., 2013; Wu et al., 2019), but also hints that many novel signals from the hypothalamus and peripheral tissues (*e.g.*, NPW, serotonin, SST, insulin, PP, and PRLH) could regulate pituitary cell functions, in which nearly all functional axes (*e.g.*, HPG, HPT, and HPA) are inter-connected. Meanwhile, our findings also strongly suggest that peripheral signals from the adipose tissue (adiponectin), gastrointestinal tract (serotonin), and pancreas (GCG, INS, SST, and PP) are likely involved in the regulation of avian pituitary functions. Taken together, avian pituitary functions are coordinately regulated by the hypothalamic inputs, peripheral signals, and local signals derived from endocrine cells (*e.g.*, GH and PRL) or non-endocrine cells (*e.g.*, WNT5A, HBEGF and activin derived from FS-cells).

### FS-Cell Is a Paracrine/Autocrine Signaling Center

**FIGURE 7:**
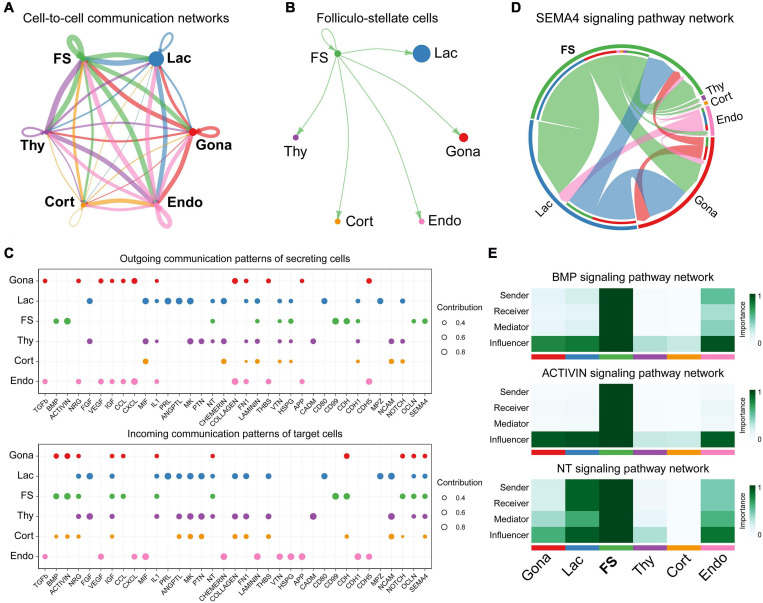
Cell-to-cell communication exploration of six chicken pituitary cell types. **(A)** Network view of cell-to-cell communications within and across six pituitary cell types. The circle sizes are proportional to the number of cells in each cell group, and edges are in proportion to the counts of ligand-receptor interaction pairs. **(B)** The inferred FS-cells signaling networks. **(C)** Dot plots showing the alteration in outgoing (ligand) or incoming (receptor) signaling pathways in pituitary cell types. The dot size is proportional to the contribution score, which is calculated from pattern recognition analysis. Higher contribution score suggests that the signaling pathway is more enriched in the corresponding cell subset. **(D)** The inferred SEMA4 signaling network. The circle segments represent the six main intercellular communication cell types with different colors. The edge width is proportional to the communication score between interacting cell clusters. **(E)** Heatmap shows the relative importance of each cell group based on the computed four network centrality measures of BMP, ACTIVIN, and NT signaling network, respectively.

To investigate the cell-cell communications among these cell subsets, we analyzed the expression levels of ligand-receptor interacting pairs within six pituitary cell types using CellChat (Jin et al., 2021). CellChat analysis detected 166 significant ligand-receptor pairs among the six cell groups, which were further categorized into 35 signaling pathways, including TGFβ, BMP, ACTIVIN, FGF, VEGF, CDH, IGF, NOTCH, SEMA4, and MIF pathways (Figure 7A). FS-cells were identified as one of the dominant communication “hubs”, which secretes and receives signals via 47 and 42 ligand-receptor pairs, respectively (Figures 7A,B). Using pattern recognition analysis, we further investigated the detailed pattern in the outgoing signaling (levels of ligands) and incoming signaling (levels of receptors) across these pathways (Figure 7C). Lactotrophs dominantly drives PRL, ANGPTL, and MK signaling, while endothelial cells drive outgoing CXCL, TGFβ, CDH5, COLLAGEN, FN1, and APP signaling. At the incoming end of signaling, gonadotrophs cells respond to ACTIVIN, NRG, CDH and NOTCH signaling; Lactotrophs and thyrotrophs cells respond to FGF, IGF, PTN, and NCAM signaling, and corticotrophs cells respond to NCAM, CADM, PTN, and MK signaling. It also revealed that FS-cells coordinate outgoing signals and incoming signals for BMP, ACTIVIN, NT (Neurotrophin), CDH, OCLN, and SEMA4 pathways (Figure 7C). This finding supports the existence of an ultra-short communication between pituitary cell populations (Schwartz, 2000; Le Tissier et al., 2012). This idea is supported by *in vitro* studies in turkeys, in which PRL can decrease LH and PRL expression and secretion in cultured pituitary cells (You et al., 1995). The reciprocal regulation or auto-regulation of endocrine cell functions adds a further level of complexity to the regulatory network of anterior pituitaries.

Network centrality analysis of the SEMA4 signaling pathway network identified that FS-cell population is the most prominent source for SEMA4 ligands acting onto gonadotrophs, lactotrophs, and thyrotrophs cells (Figure 7D). Interestingly, CellChat also predicted that FS-cell population significantly contributes to dominated BMP and ACTIVIN signal production in the pituitary, and FS-cells are the primary NTF3 ligand source, which mainly acts onto lactotrophs and endothelial cell populations, in which *NTRK3* was highly expressed (Figure 7E).

**FIGURE 8:**
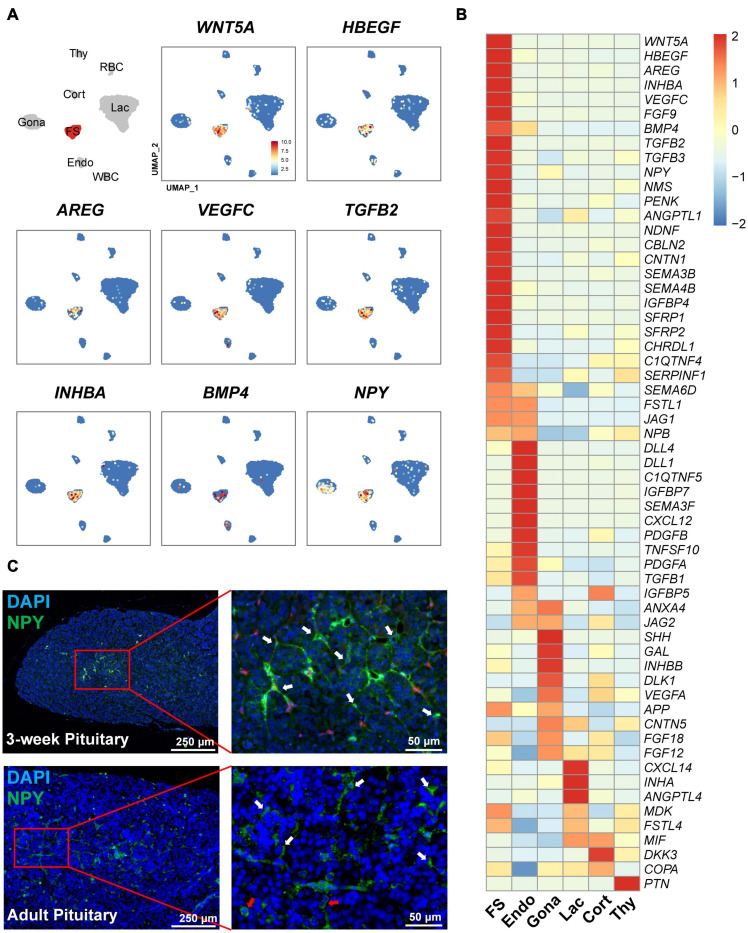
Expression of the genes encoding growth factors and peptides in FS-cells and other cell clusters. **(A)** UMAP maps showing the abundant/specific expression of some genes encoding growth factors and peptides (*WNT5A, HBEGF, AREG, VEGFC, TGF*β*2*, activin A (encoded by *INHBA*), *BMP4*, and *NPY*). Dark red and light blue indicate high and low expression levels, respectively. **(B)** Heat-map showing the mRNA levels of the genes encoding growth factors and peptides in FS-cells and other cell types. The expression level of each gene was normalized to the *z*-score and color-coded. **(C)** Validation of neuropeptide Y (NPY) expression in FS-cells (marked by white arrows) and endocrine cells (red arrows) of 3-week chick and adult chicken anterior pituitaries by immunofluorescent staining. The NPY immuno-reactive signal was labeled with Alexa Fluor 488 (green). Nuclei are stained with DAPI (blue). Scale bar: 250 μm/50 μm.

Interestingly, we also found a list of genes encoding growth factors and peptides abundantly expressed in this FS-cell cluster by heatmap analysis (Figure 8). These genes include semaphorin 3B (*SEMA3B*), *SEMA4B, BMP4, INHBA* (producing activin A), *WNT5A*, heparin-binding EGF-like growth factor (*HBEGF*), aphiregulin (*AREG*) (Wang et al., 2007), vascular endothelial growth factor C (*VEGFC*), transforming growth factor β2 (*TGFB2*), *TGFB3*, and neuropeptide Y (*NPY*) (Gao et al., 2017), suggesting that unlike other pituitary cell types (Figure 8), FS-cells can produce a number of growth factors and peptides.

In line with scRNA-seq analysis showing *NPY* expression in FS-cell (and Gona-) cluster, NPY-immunoreactive (NPY-ir) signals were likely localized in ‘star/stellate-shape’ FS-cells and some endocrine cells distributed within the pituitary cephalic lobe and caudal lobe, which resembles the distribution of LH-cells (Proudman et al., 1999). This finding also supports the reliability of scRNA-sequencing results (Figure 8C).

Apart from genes encoding growth factors/peptides, many genes encoding transcription factors, WNT receptors, Shh signaling components, the pathway inhibitors of WNT, BMP and IGFs signaling (Supplementary Figure 4), and proteins associated with the extracellular organization (Supplementary Figure 5), cell-cell junction (Supplementary Figure 6), biological oxidation (Supplementary Figure 7), fatty acid metabolism (Supplementary Figure 8), steroid metabolism (Supplementary Figure 9), cholesterol biosynthesis (Supplementary Figure 10), peptide/protein processing and epithelial-to-mesenchymal transition (EMT) (Supplementary Figure 11), have also been found to be expressed in FS-cells. Moreover, we noted that the mRNA levels of the afore-mentioned genes differ from those in all other pituitary cell types (Supplementary Figures 4–11).

It has been hypothesized that in mammals, FS-cell may regulate endocrine cell functions by secretion of paracrine/autocrine factors, such as VEGF, annexin 1 (AXNA1), follistatin, PACAP, TGFβ1, WNT5A, and leptin (LEP) (Denef, 2008). However, this hypothesis still lacks experimental support from other model animals. In this study, we found that FS-cells can abundantly or specifically express many genes encoding growth factors and peptides (Figure 8). Moreover, the cognate receptors for most of these factors/peptides were found to be expressed in other cell populations. In agree with the finding in cell-cell communications by CellChat, our findings strongly suggest that FS-cells are capable of producing a list of growth factors/peptides (Figure 8), and thus likely act as a paracrine/autocrine signaling center to control the proliferation, differentiation, apoptosis and functions of pituitary cells. For instance, activin A (a homodimer of activin βA subunit) from FS-cells may stimulate *FSHB* expression in gonadotrophs expressing activin receptors (ACVRs), as demonstrated in mammals and teleosts (Gregory et al., 2005; Lau and Ge, 2005), while HBEGF/AREG/FGFs from FS-cells may stimulate cell proliferation, or prevent cell apoptosis/differentiation via EGFR/FGFR family members, as documented in various normal/cancerous tissues (Normanno et al., 2006; Wang et al., 2007; Tiong et al., 2013).

Besides the growth factors/peptides, we also found the abundant expression of *ALDH1A3* and *ALDH1A1*, the two enzymes for retinoic acid (RA) synthesis, in FS-cells. This finding points to a possibility that RA is likely produced in the FS-cells and acts as an additional paracrine/autocrine factor to regulate the differentiation and function of cells expressing RA receptors (RARs)/rational X receptors (RXRs) (Figure 6), similar to that proposed in rat anterior pituitary (Maliza et al., 2017). Interestingly, the antagonists or inhibitors for WNT (*SRFP1, SRFP2*), BMP (*FSTL1, CHRDL*), and IGF1 (*IGFBP4*) signaling were also expressed in FS-cells. These findings suggest their involvement in fine-tuning the paracrine/autocrine signaling of WNTs/BMPs/IGFs in responsive cells (Figures 7C, 8).

In this study, we also noted that many receptors are expressed in FS-cells, such as SHH receptor (PTCH1), PRLR, and activin/TGFβ/BMP receptors (Figure 6). Since *SHH* is highly expressed in gonadotrophs (Supplementary Figure 4), and *PRL* is predominantly expressed in lactotrophs, respectively, our finding strongly suggests that FS-cells can also receive signals from neighboring cells, including endocrine cells and FS-cells. This ‘bi-directional cell-to-cell communication’ may be crucial for dynamic control of pituitary cell composition and function to meet the physiological demands in chickens, as reported in mammals (Denef, 2008; Ren et al., 2019).

### FS-Cell May Enrich Progenitor/Stem Cells

**FIGURE 9:**
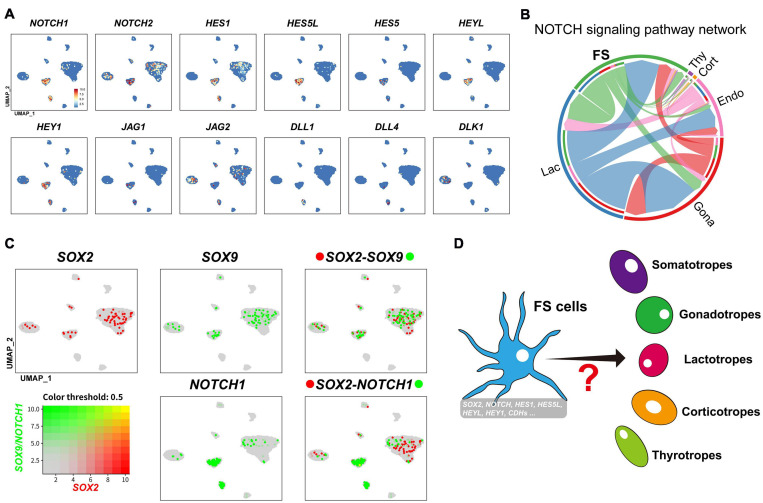
**(A)** UMAP maps showing the abundant/specific expression of genes encoding Notch signaling components (*NOTCH1, NOTCH2, HES1, HES5L, HES5, HEYL*, and *HEY1*) in FS-cell cluster and Notch ligands (*JAG1, JAG2, DLL1, DLL4*, and *DLK1*) in FS-cells, endothelial cells, and endocrine cell clusters (Gona and Lac). Dark red and light blue indicate high and low expression levels, respectively. **(B)** The intercellular communication network of NOTCH signaling pathway. The circle segments represent the six main intercellular communication cell types with different colors. The edge width is proportional to the communication score between interacting cell clusters. **(C)** Co-expression analysis of *SOX2* and *SOX9* in chicken pituitary FS-cells and other cell clusters. Cells abundantly co-expressing *SOX2* and *SOX9* are shown in yellow. **(D)** A simplified model showing that a sub-population of FS-cell cluster co-expressing many progenitor/stem cell-associated factors previously reported (Notch signaling components, *CDH1* and *SOX2 etc.* (Vankelecom, 2010; Edwards and Raetzman, 2018) may represent the progenitor/stem cell sub-population, which could differentiate into endocrine cells (*e.g.*, lactotrophs and gonadotrophs) under some unknown induction signals in adult pituitary (marked by question mark).

Interestingly, in the FS-cell cluster, we also observed a relatively high expression of NOTCH signaling components (*NOTCH1, NOTCH2, HEY1, HEYL, HES1, HES5*, and *HES5-like* (*HES5L, ENSGALG00000001136*). This finding, together with the expression of Notch ligands (*JAG1, JAG2, DLL1, DLL4*, and *DLK1*) in neighboring cells, support the existence of NOTCH signaling in FS sub-population (Figures 7C,  9A,B).

It was proposed that FS-cells may be one source of progenitor/stem cells in mammals, however, this hypothesis has received little attention to date (Inoue et al., 2002; Vankelecom, 2007). In this study, we found that chicken FS-cells can express many transcription factors (*e.g., Pitx1, Pitx2, Lhx3, PRRX1*, and *PRRX2*), which resembles the characteristics of embryonic pituitary stem/progenitors (Supplementary Figure 4; Kelberman et al., 2009). Moreover, FS-cells can also express a set of NOTCH signaling components, which have been shown to be crucial for the maintenance, expansion, and cell-fate choice of progenitor/stem cells of the anterior pituitary and other tissues in mammals (Suh et al., 2002; Edwards and Raetzman, 2018). Since NOTCH ligands could be identified in neighboring cells, such as gonadotrophs (JAG2, DLK1), lactotrophs (JAG2), endothelial cells (DLL1 and DLL4), and FS-cells (JAG1) (Figures 8, 9), this supports the existence of juxtracrine NOTCH signaling in chicken pituitary FS-cells. In addition, many genes associated with mammalian stem cell niche formation (E-cadherin) (Batchuluun et al., 2017), or the stem cell renewal, survival, and fate determination of the pituitary and other tissues (*e.g.*, PAX6, ID1-4, Shh-PTCH signaling, WNT signaling, EGFR and FGFR signaling, ALDH1A1, and ALDH1A3) (Ying et al., 2003; Chen et al., 2005; Fauquier et al., 2008; Jiang et al., 2008; Sansom et al., 2009; Vankelecom and Chen, 2014; Zhu et al., 2015; Tomita et al., 2016), are abundantly expressed in chicken FS-cells. The co-expression of so many progenitor/stem cell-associated genes (shown in Supplementary Figure 11) in the FS-cell cluster (Vankelecom, 2010; Yavropoulou et al., 2015) led us to propose that adult progenitor/stem cells, resembling the features of embryonic progenitor/stem cells, may be enriched in the FS sub-population, and thereby confer some plasticity to meet the physiological needs in adult pituitary. Clearly, more studies are required to substantiate this hypothesis.

As the pituitary progenitor/stem-cell markers reported in mammals (Fauquier et al., 2008), *SOX2* was also found to be expressed in the FS-cell and endocrine cells (*e.g.*, gonadotroph) of chicken pituitaries (Figure 9B and Supplementary Figure 12). In mammals, SOX2/SOX9-positive cells have been suggested to be pituitary progenitor/stem cells (Yoshida et al., 2011; Andoniadou et al., 2013). In this study, we found that *SOX2*/*SOX9* is expressed in FS-cells and other endocrine cells, such as in lactotrophs and gonadotrophs abundantly expressing *PRL* and *LHB*, respectively (Figure 9B and Supplementary Figure 12). The expression of *SOX2*/*SOX9* in fully differentiated endocrine cells indicates that unlike that in mammals (Cheung et al., 2018), SOX2 may not be the sole marker for adult pituitary progenitor/stem cells in chickens. Therefore, future study focused on FS sub-population co-expressing the set of progenitor/stem cell-associated genes (NOTCH signaling molecules, *CDH1, SOX2*, and *Pitx2 etc.*) (Supplementary Figure 12) may help to precisely define the avian pituitary progenitor/stem cells, and thereby, allow us to trace their differentiation routes, when signaled by inner physiological demands and environmental cues (*e.g.*, light, temperature, food availability) (Figure 9C).

### Sexual Dimorphism of Gene Expression Pattern in Pituitary Cell Clusters

**FIGURE 10:**
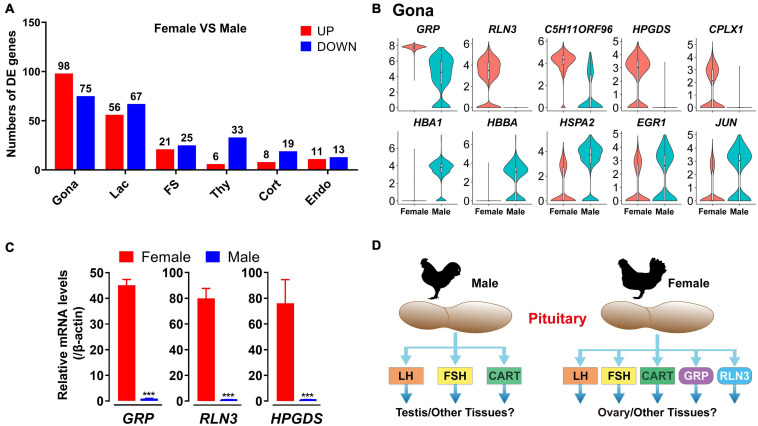
Sexually dimorphic expression of genes in adult chicken male and female anterior pituitary cell clusters. **(A)** Bar charts presenting the numbers of differentially expressed (DE) genes in each cell cluster in male and female chicken anterior pituitaries. Differences in gene expression level were significant if the adjusted *P*-value was below the multiple-testing threshold of 0.001 and the absolute log2 expression fold change was above 0.5; **(B)** Violin plots showing the normalized expression levels of selected genes differentially expressed in gonadotrophs (Gona) of male and female chicken pituitaries. Box plot: median (center black line), interquartile range (white box) and minimum-maximum range (whiskers). **(C)** qPCR detection of chicken *GRP, RLN3* and *HPGDS* mRNA levels between male and female chicken pituitaries. Each data point represents the mean ± SEM of four laying hens/male chickens (*N* = 4). ***, *P* < 0.001 between two groups. **(D)** Major endocrine hormones secreted by pituitary gonadotrophs of adult male and female chickens. In male chickens, gonadotroph can abundantly express *LHB, FSHB* and *CART*, and thus may produce three major endocrine hormones, which may play important roles in the testis (and other tissues), whereas in hens, gonadotrophs may produce 5 major hormones (LH, FSH, CART, GRP, and RLN3) and likely play roles in the ovary (and other tissues). However, this hypothesis needs further verification.

In this study, sexually dimorphic expression of many genes in endocrine cell, FS-cell and Endo-cell clusters have been found, particularly, in gonadotroph (173 genes), lactotroph (123 genes) and FS-cell (46 genes) clusters (Figure 10A and Supplementary Data 2). More than 98 genes including *RLN3, GRP, C5H11ORF96, CPLX1* and *HPDGS* are shown to be specifically/preferentially expressed in gonadotrophs of female chicken anterior pituitaries, while 75 genes including *HBA1, HBBA, HSPA2, EGR1*, and *JUN*, are found to be expressed in the male gonadotroph cluster specifically/preferentially (Figure 10B and Supplementary Data 2). Using quantitative real-time PCR (qPCR), we also confirmed the sexually dimorphic expression patterns of *GRP, RLN3*, and *HPGDS* at the whole anterior pituitary level (Figure 10C).

Marked sexual dimorphism was also evident in endocrine cell clusters, particularly in lactotrophs and gonadotrophs, in rodents (Fletcher et al., 2019; Ho et al., 2020). The predominant expression of *FSHB* mRNA in male chicken gonadotrophs was consistent with previously reports in rodents. These findings, taken together, support a functional difference of the anterior pituitary between the two sexes in birds and mammals. We noted that some genes with dimorphic expression are associated with their localization on sex chromosomes Z (18 genes, including *RLN3, GRP, CPLX1, HEXB, FAM189A2 etc.*) or W (2 genes, *HINTW* and *ENSGALG00000040263*) (Supplementary Data 2). However, most genes with sexually dimorphic expression are localized on autosomes, which expression is likely controlled by sex-dependent factors, such as sex steroids.

The sexually dimorphic expression patterns of genes in pituitary cells may have important physiological relevance. As an example, novel peptide hormones GRP and RLN3, highly expressed in female (and not male) chicken pituitary gonadotrophs, may play important roles in female reproduction, such as ovary/oviduct development and functions (Figure 10D). Future studies on these genes in chickens and other birds not only can help to uncover their influence on growth, metabolism, stress, and reproduction of male and female birds, but also help to reveal their association with the phenotypic traits of birds (*e.g.*, egg-laying performance, reproduction mode, meat production, body composition, and behaviors) (Fallahshahroudi et al., 2019).

## Conclusion

In summary, we identified four endocrine and four non-endocrine cell population within the chicken pituitary and characterized their gene expression profiles, including their sexually dimorphic gene expression. We also found several inter-cellular crosstalk between the cell clusters in adult chickens (Figure 7A). Among these cell types, FS-cells can express many genes encoding growth factors and peptides and they likely act as a paracrine/autocrine signaling center to influence neighboring cells. Undoubtedly, our data sets a critical reference point to reveal the novel aspects of pituitary biology across vertebrates, and helps to figure out how the anterior pituitary, as a pivotal signal converging and output center for the brain and peripheral tissues, can orchestrate so many physiological processes (*e.g.*, growth, reproduction, metabolism, and stress) to meet the changing physiological demands or pathological status observed in vertebrates, including birds and humans.

## Data Availability Statement

The scRNA-seq clean data reported in this paper have been deposited in the Genome Sequence Archive in National Genomics Data Center, China National Center for Bioinformation/Beijing Institute of Genomics, Chinese Academy of Sciences, under accession number CRA003604 that are publicly accessible at https://ngdc.cncb.ac.cn/gsa/.

## Ethics Statement

All animal experiments were conducted in accordance with the Guidelines for Experimental Animals issued by the Ministry of Science and Technology of People’s Republic of China. All animal experimental protocols were approved by the Animal Ethics Committee of the College of Life Sciences, Sichuan University (Chengdu, China).

## Author Contributions

JZ did the investigation, data analysis, writing-original draft, and writing-review and editing. CL did the conceptualization, investigation, and writing-original draft. CM did the conceptualization and investigation. ML and YPW did the investigation. JL did the resources and writing-review and editing. YJW did the conceptualization, resources, writing-original draft, and writing-review and editing. All authors contributed to the article and approved the submitted version.

## Conflict of Interest

The authors declare that the research was conducted in the absence of any commercial or financial relationships that could be construed as a potential conflict of interest.

## Footnotes

1 http://www.ensembl.org/Gallus_gallus

2 ftp://ftp.ensembl.org/pub/release-72/fasta/gallus_gallus/dna
