# Supplementary material for: Single-Cell RNA Sequencing Analysis of Chicken Anterior Pituitary: A Bird’s-Eye View on Vertebrate Pituitary
Source: Front Physiol. 2021 Jun 29;12:562817. doi: 10.3389/fphys.2021.562817 (PMC8276247; doi:10.3389/fphys.2021.562817)
Supplement: Supplementary file 1 [file Data_Sheet_1.docx]

**Supplementary Information**

**Title:**

**Single-cell RNA Sequencing Analysis of Chicken Anterior Pituitary: A bird’s-eye View on Vertebrate Pituitary**

**Authors:**

**Jianan Zhang, Can Lv, Chunheng Mo, Meng Liu, Yiping Wan, Juan Li, Yajun Wang**

**Table S1. Primers used for qRT-PCR assay of sexually dimorphic expression of genes in chicken anterior pituitary**

| Gene | Sense/antisense | Primer sequence (5’-3’) | Size (bp) |
| --- | --- | --- | --- |
| *GRP* | Sense | CCCGCGCTCACCAAGATCTA | 128 |
|  | Antisense | GGTGATGCTGACAATGGGATCT |  |
| *RLN3* | Sense | ATTCTTCTCAAGCAGCAAGT | 169 |
|  | Antisense | TCTTTGAAGTCATCTGCCAT |  |
| *HPGDS* | Sense | AGGACCCATCATGCCCAACT | 189 |
|  | Antisense | ATCACTCCATCTACTTCCAG |  |
| *β-actin* | Sense | CCCAGACATCAGGGTGTGATG | 123 |
|  | Antisense | GTTGGTGACAATACCGTGTTCAAT |  |

**Supplementary Data 1**

**The full list of marker genes identified in chicken anterior pituitary cell cluster**

**Supplementary Data 2**

**Sexually dimorphic expression of genes in seven pituitary cell clusters of adult anterior pituitary**

B

A


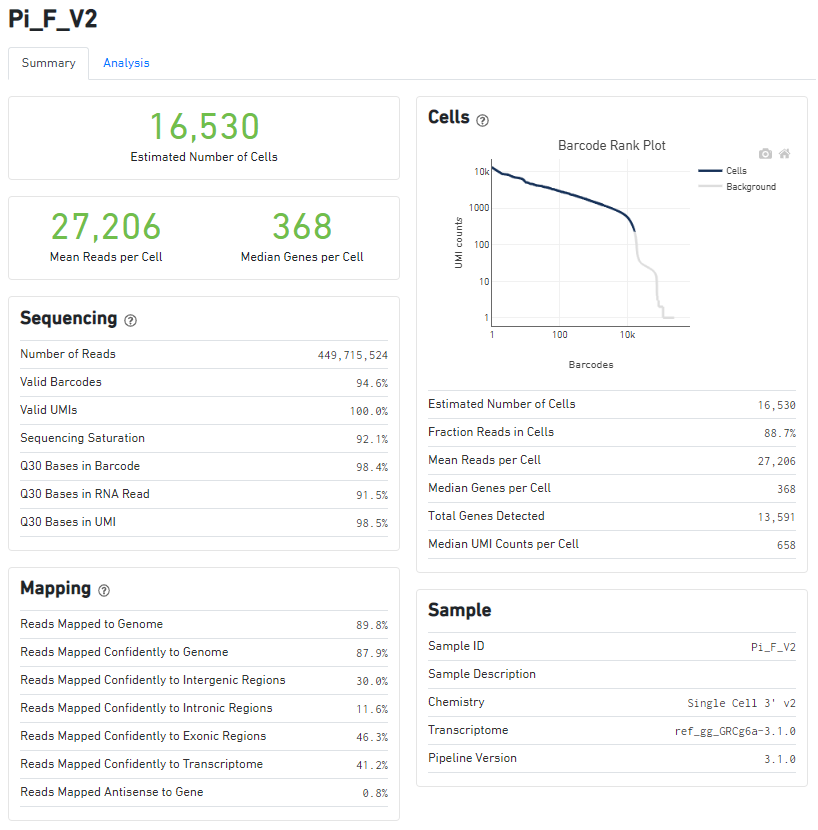

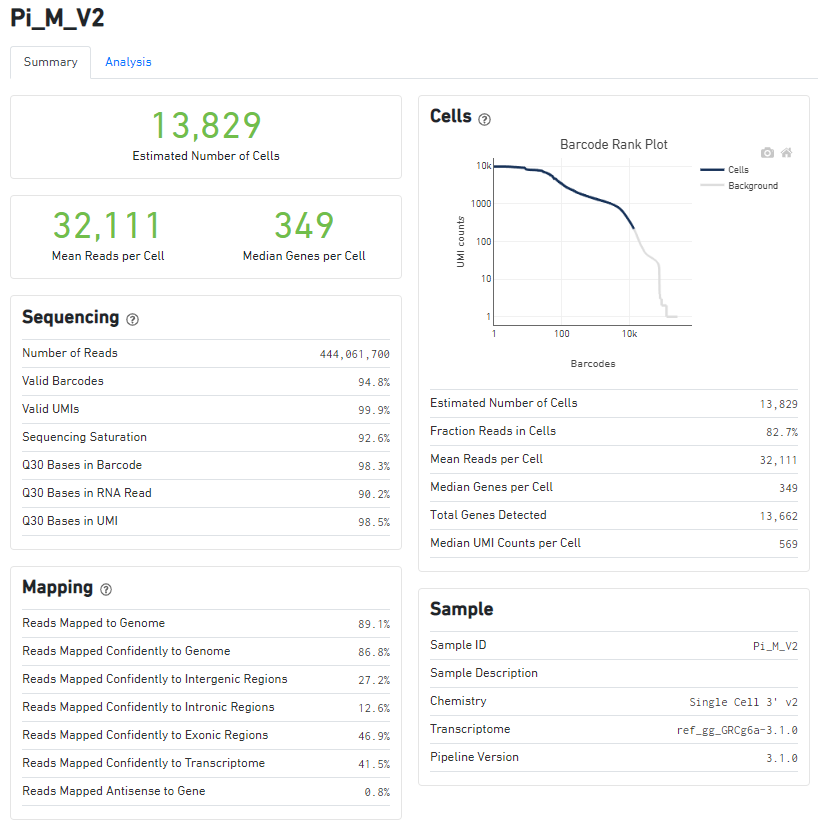


C


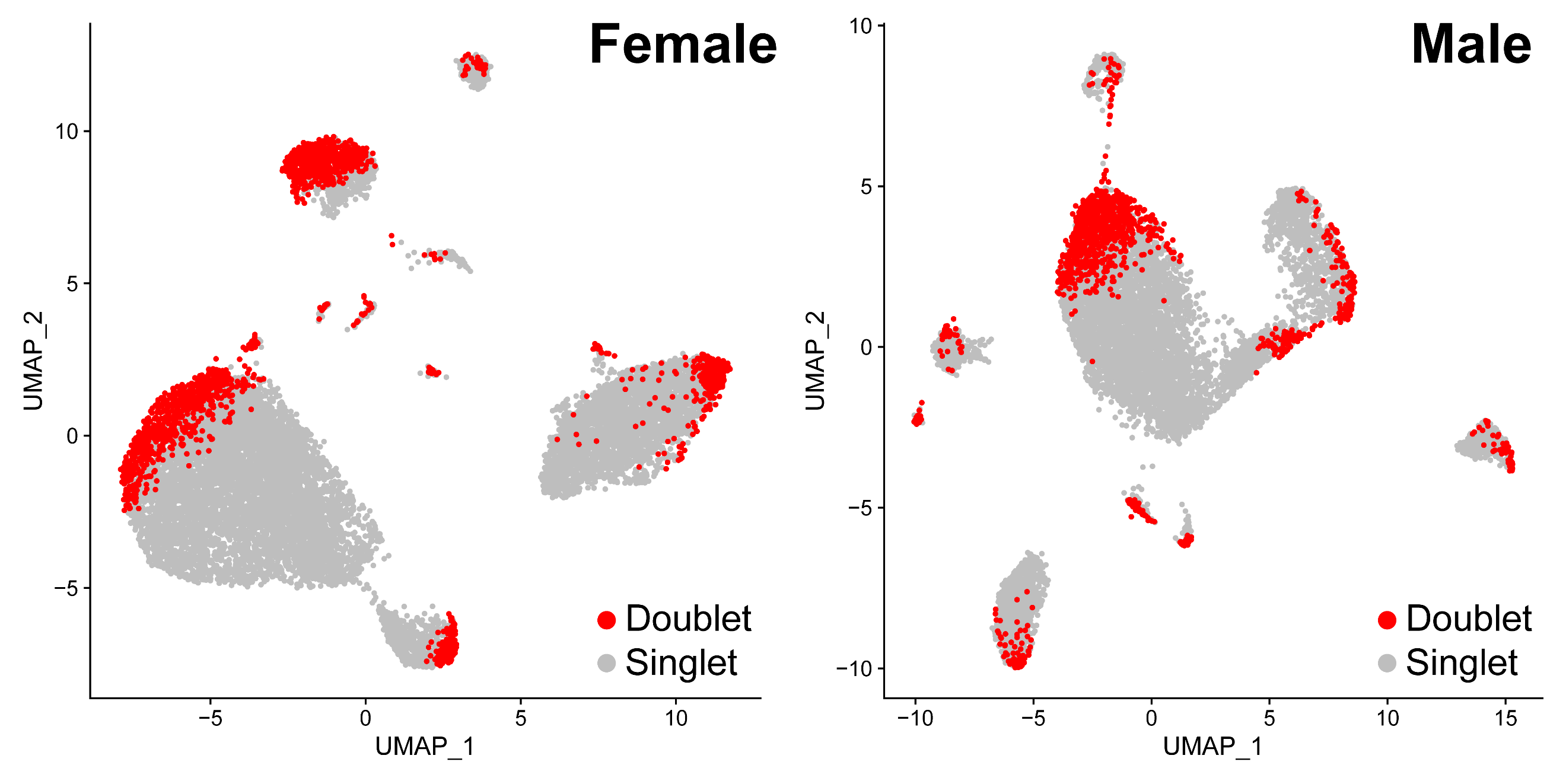


**Figure S1.** (A-B)10x Genomics Cell Ranger software summaries of unfiltered data from female (Pi_F_V2, A) and male (Pi_M_V2, B) chicken anterior pituitary samples. Six anterior pituitaries from each sex were collected to prepare the dispersed pituitary cell used for scRNA-sequencing. (C) Combined Uniform Manifold Approximation and Projection (UMAP) plot annotated by output of DoubletFinder analysis. We used DoubletFinder to detect 15% likely doublet cells that we subsequently discarded.


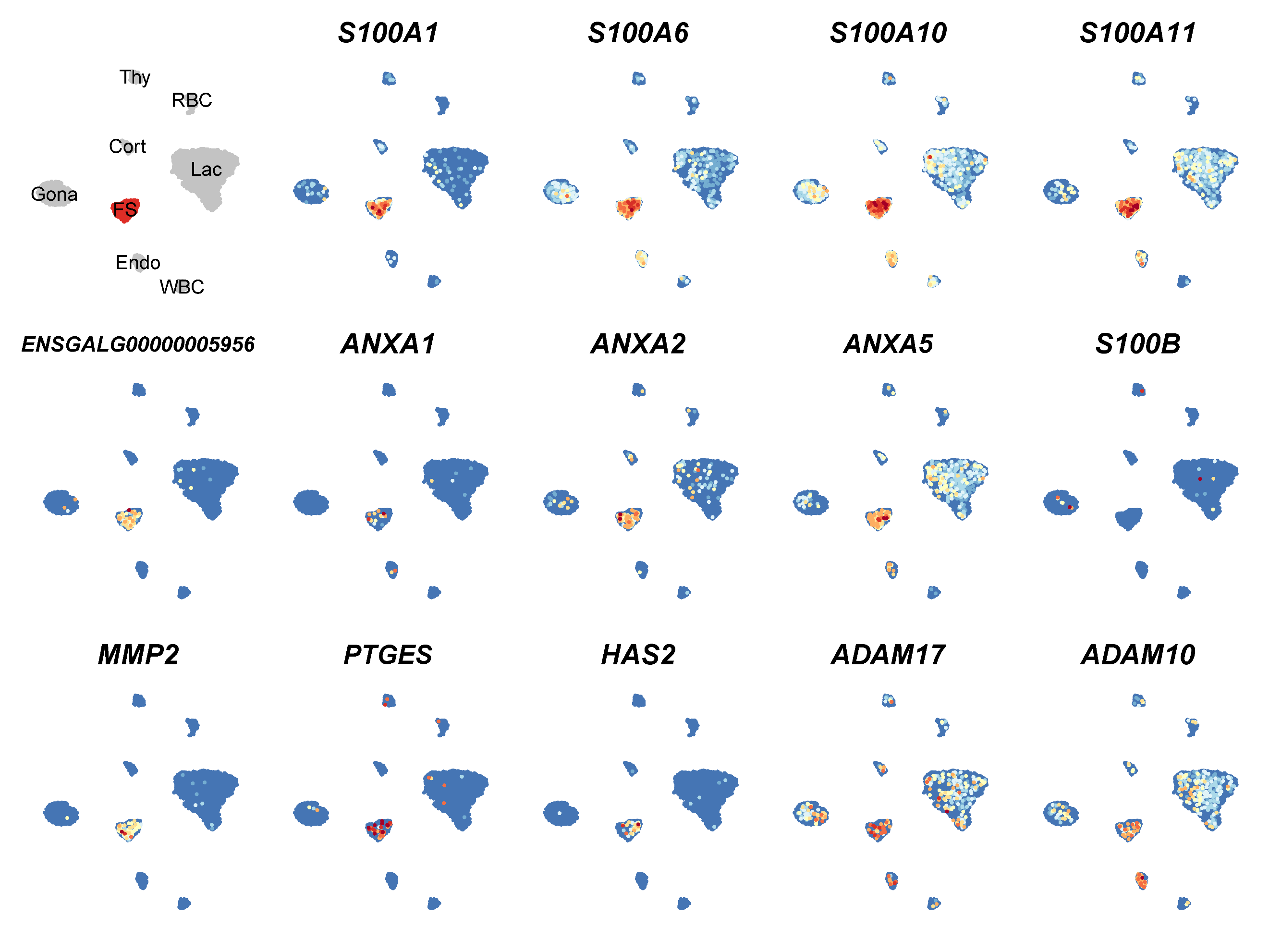


A

B

**Figure S2. (A)** UMAP maps showing the expression of some marker genes (*S100A1, S100A6, S100A10, S100A11, ENSGALG00000005956(ANXA5-like), ANXA1, ANXA2, ANXA5*) expressed in FS-cell cluster of chicken anterior pituitary. Unlike that in mammals, *S100B* seems not to be expressed in FS-cell cluster of chicken anterior pituitary, specially. **(B)** UMAP maps showing the expression of some genes encoding enzymes (*MMP2, PTGES, HAS2, ADAM17,* and *ADAM10*) expressed in FS-cell cluster of chicken anterior pituitary.


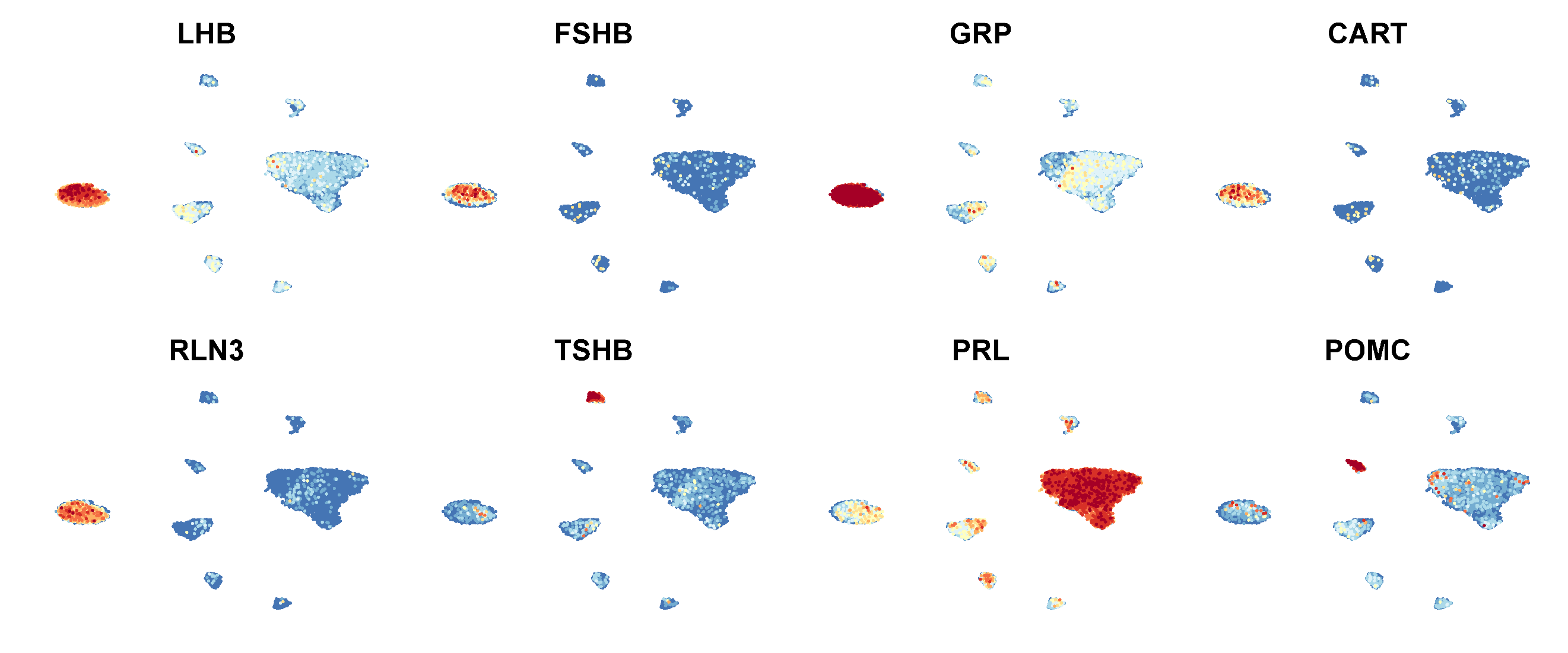


**Figure S3.** UMAP maps showing the expression of pituitary hormone genes (e.g., *LHB, FSHB, GRP, CART, GH, TSHB, PRL, POMC*) are expressed in multiple cell types. *LHB, FSHB, GRP, CART, GH, TSHB, PRL,* and *POMC* are predominantly expressed in one endocrine cell types (marked by dark red), however, they are also weakly expressed in other endocrine cell type(s).


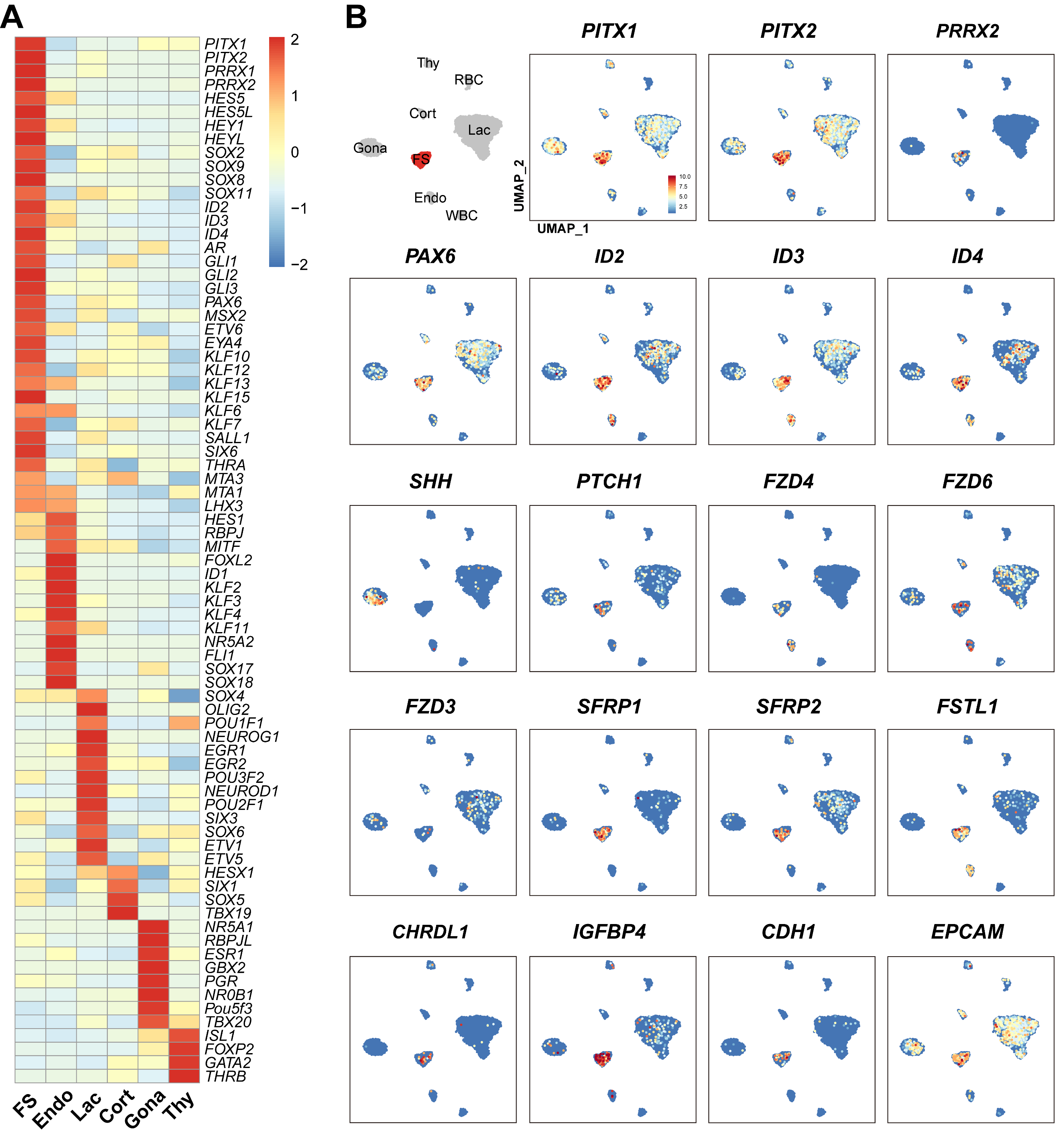


**Figure S4.** Heat-map showing the expression of genes encoding the transcription factors in FS-cells and other cell types indicated, including those crucial for embryonic pituitary organogenesis and progenitor cell specification/terminal differentiation. The expression level of each gene was normalized to the z-score and color-coded. (B) UMAP maps showing the expression of transcription factors (*PITX1*, *PITX2*, *PRRX2*, *PAX6*, *ID2*, *ID3*, *ID4*), *SHH*, *PTCH1*, WNT receptors (*FZD4*, *FZD6*, *FZD3*), binding proteins for WNT (*SFRP1*, *SFRP2*), BMPs (*FSTL1*, *CHRDL1*) and IGF (*IGFBP4*), and adhesion molecules (*CDH1* and *EPCAM*) in FS-cells and endocrine cells. Dark red and light blue indicate high and low expression levels, respectively.


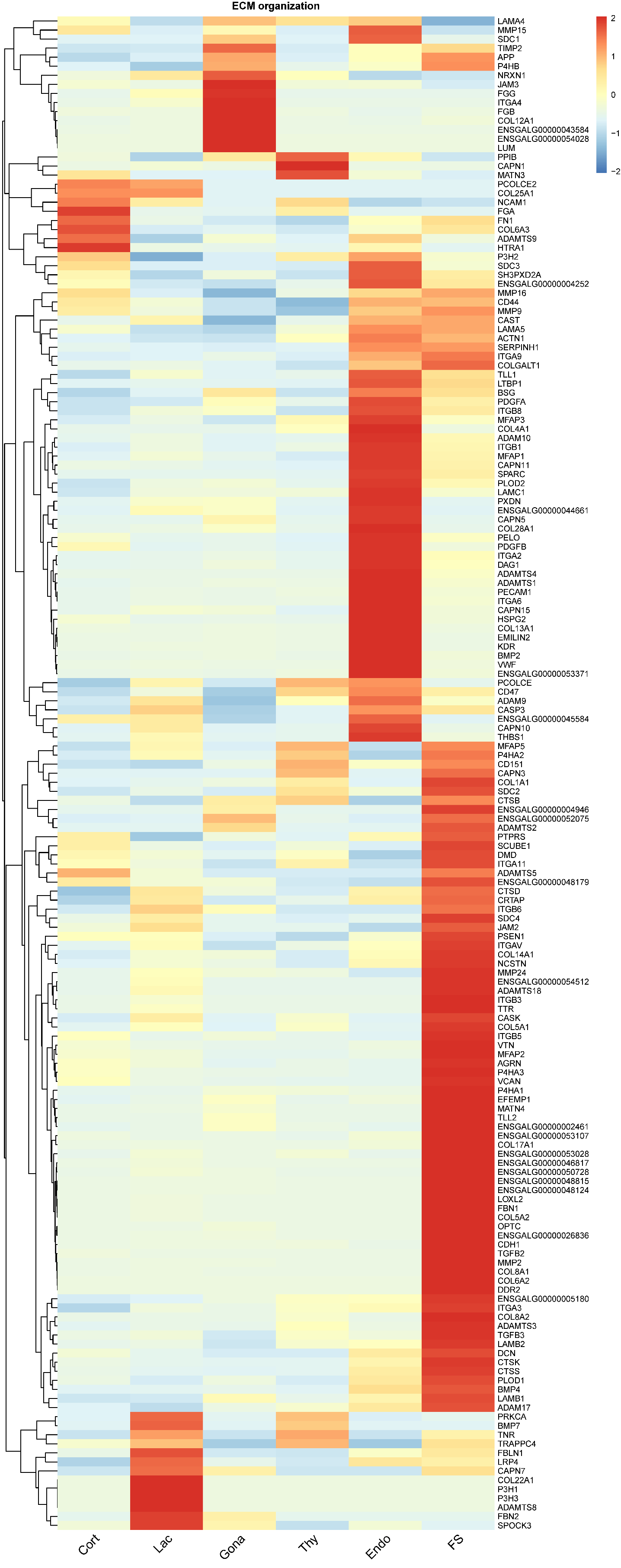


**Figure S5.** Heat-map showing expression level of genes associated with extracellular matrix (ECM) organization across different cell types.

A


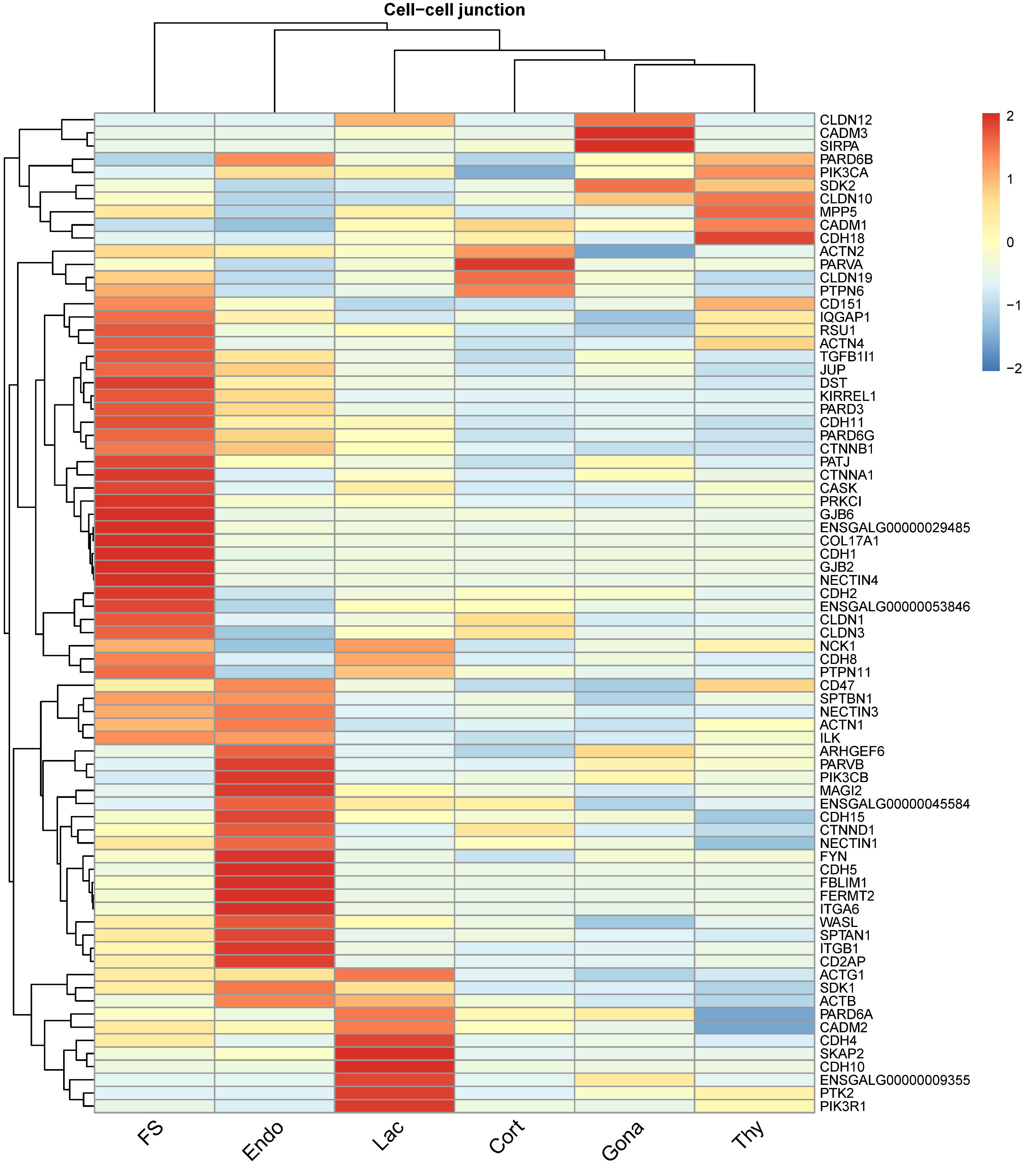


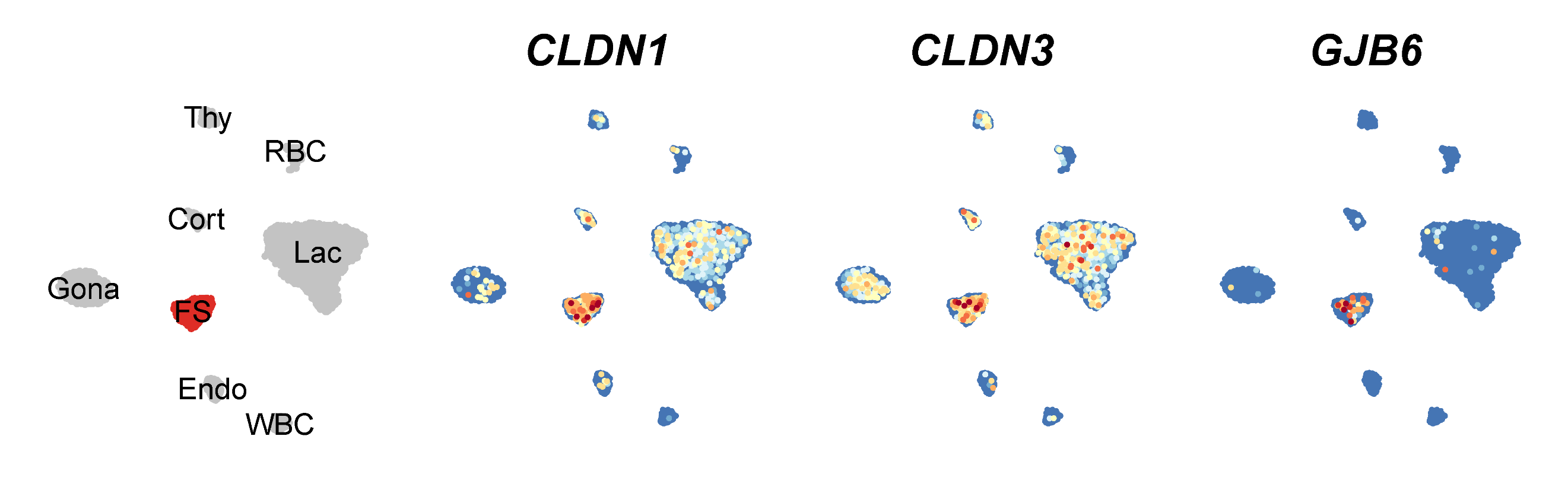


B

**Figure S6.** (A) Heat-map showing expression level of some genes encoding proteins associated with cell-cell junction across different cell types. Gene expression levels are color coded. The expression level of each gene was normalized to the z-score and color-coded. (B) UMAP maps showing the expression of some genes for tight junction (*CLDN1*, *CLDN3*) and gap junction (*GJB6*) expressed in FS cluster of chicken anterior pituitary.


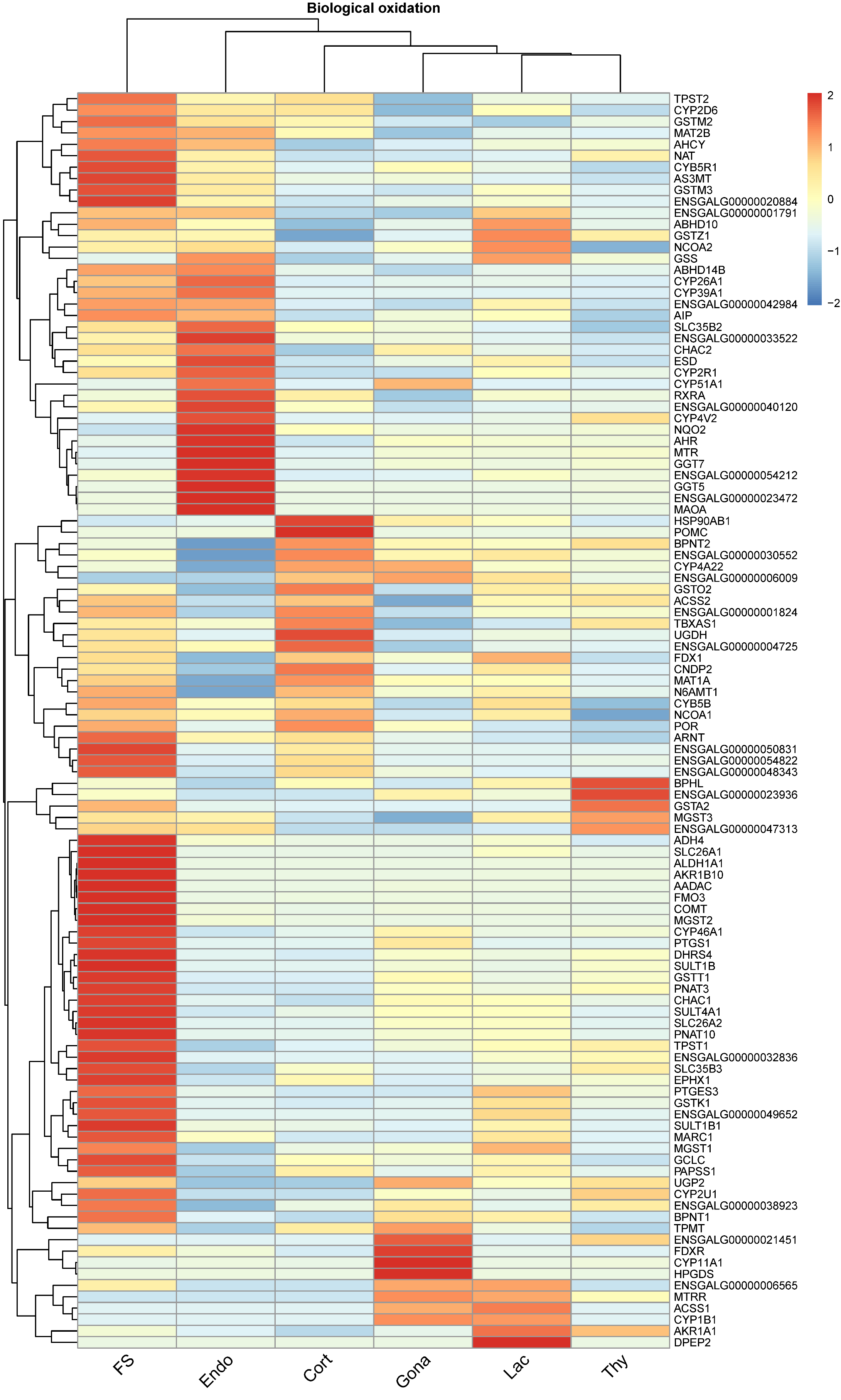


**Figure S7.** Heat-map showing expression level of some genes encoding proteins associated with biological oxidation across different cell types. Gene expression levels are color coded. The expression level of each gene was normalized to the z-score and color-coded.


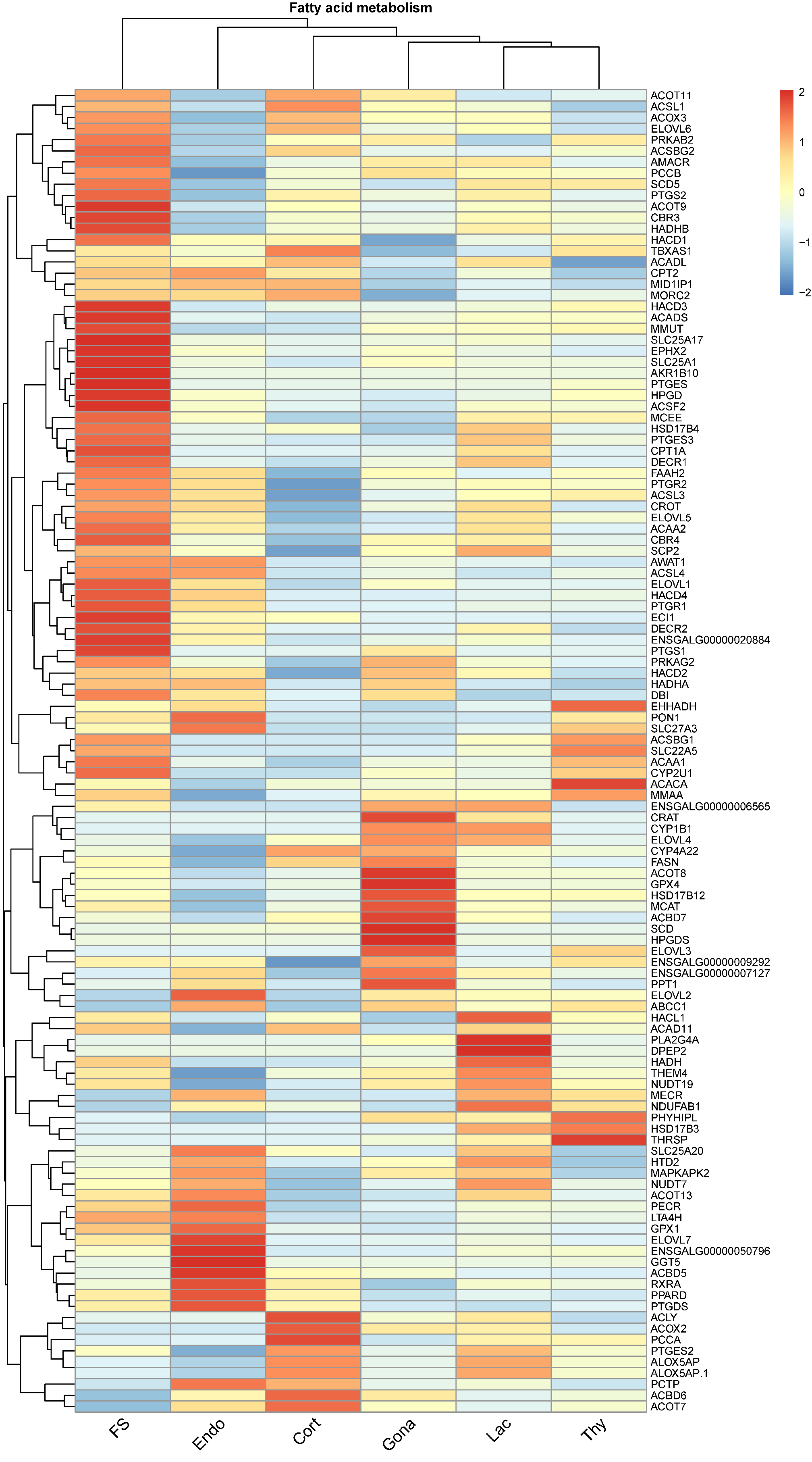


**Figure S8.** Heat-map showing expression level of some genes associated with fatty acid metabolism across different cell types. Gene expression levels are color coded. The expression level of each gene was normalized to the z-score and color-coded.


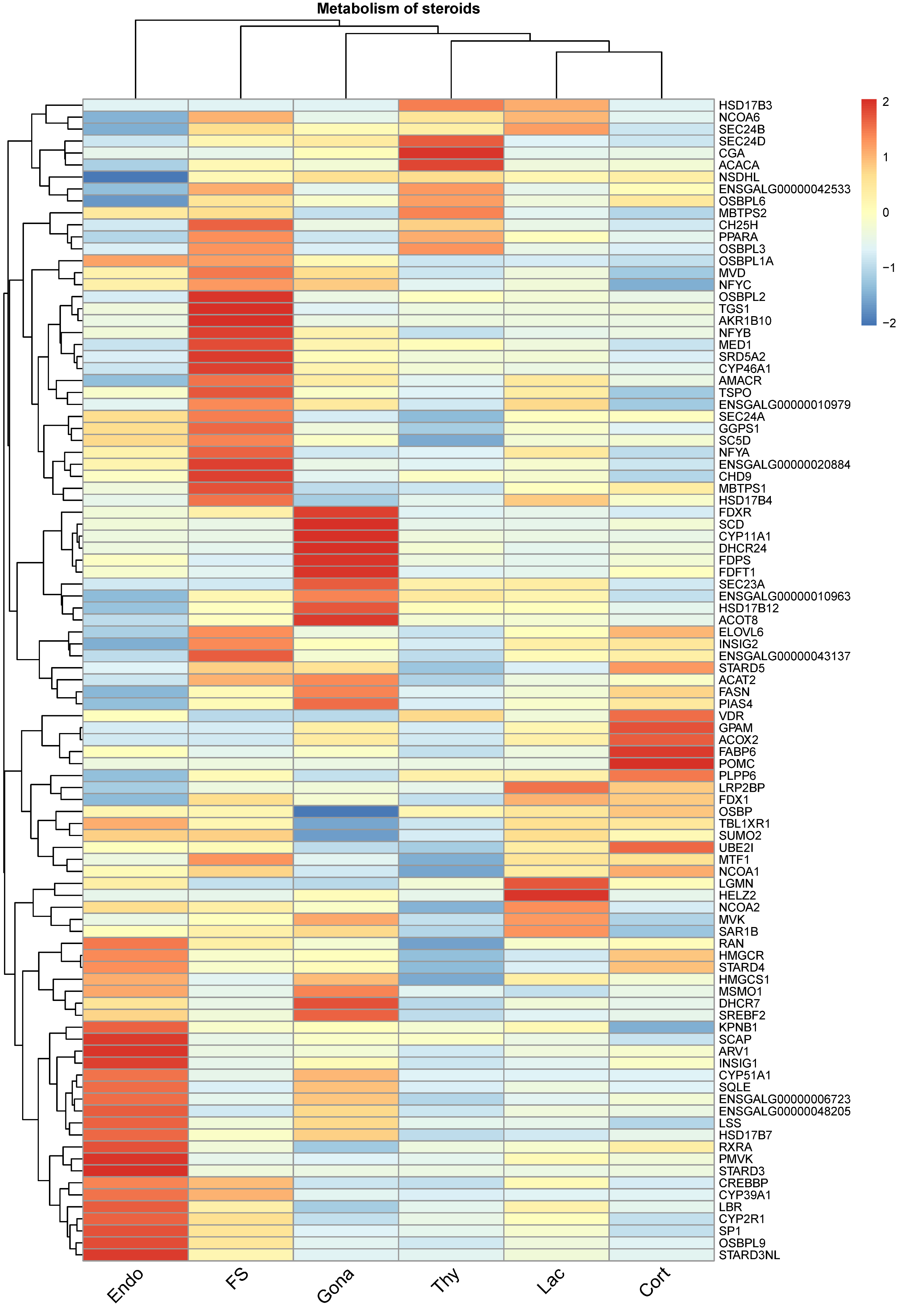


**Figure S9.** Heat-map showing expression level of some genes associated with metabolism of steroids across different cell types. Gene expression levels are color coded. The expression level of each gene was normalized to the z-score and color-coded.


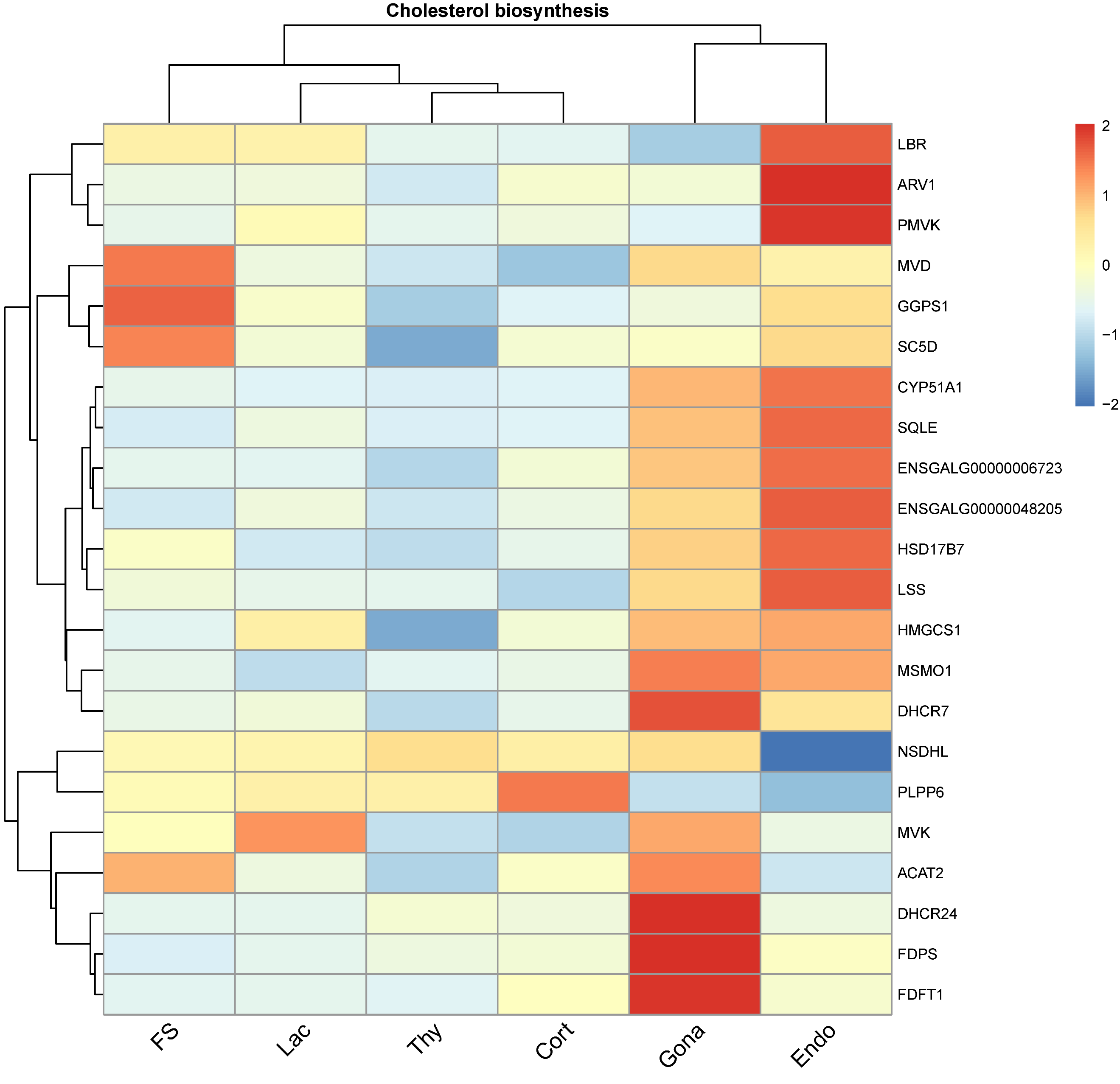


**Figure S10.** Heat-map showing expression level of some genes associated with cholesterol biosynthesis across different cell types. Gene expression levels are color coded. The expression level of each gene was normalized to the z-score and color-coded.


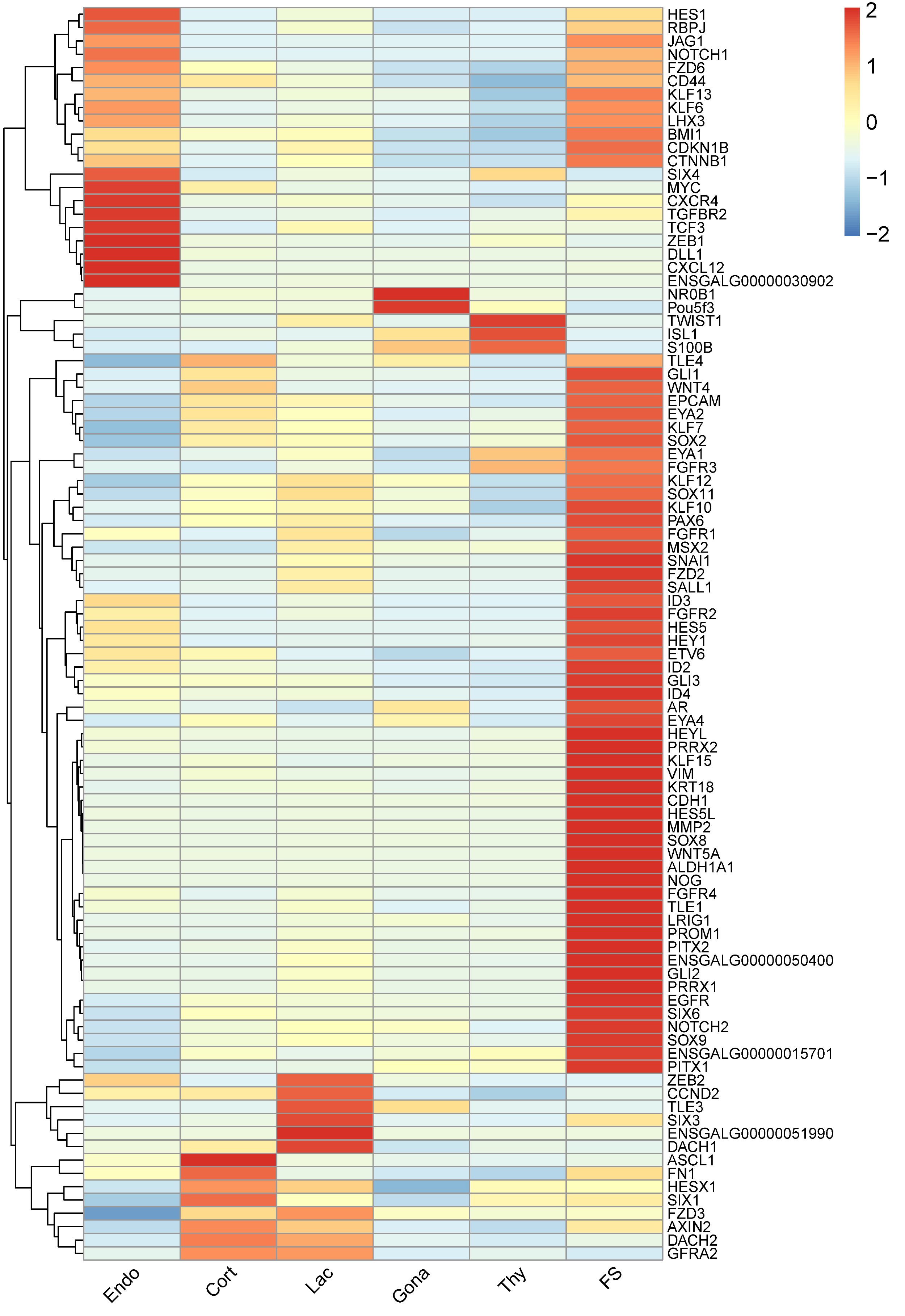


**Figure S11.** Heat-map showing expression level of some potential genes [such as the transcription factors (Pitx2, ID1-4, and PAX6) identified in embryonic pituitary progenitors, cell adhesion molecules, signaling molecules of Notch, WNTs, SHH-PTCH1, FGFR and EGFR, and molecules (e.g., VIM) of epithelial-to-mesenchymal transition (EMT)] associated with stem/progenitor cells across different cell types, which have been reported in mammalian pituitary or non-pituitary tissues. Gene expression levels are color coded. The expression level of each gene was normalized to the z-score and color-coded.


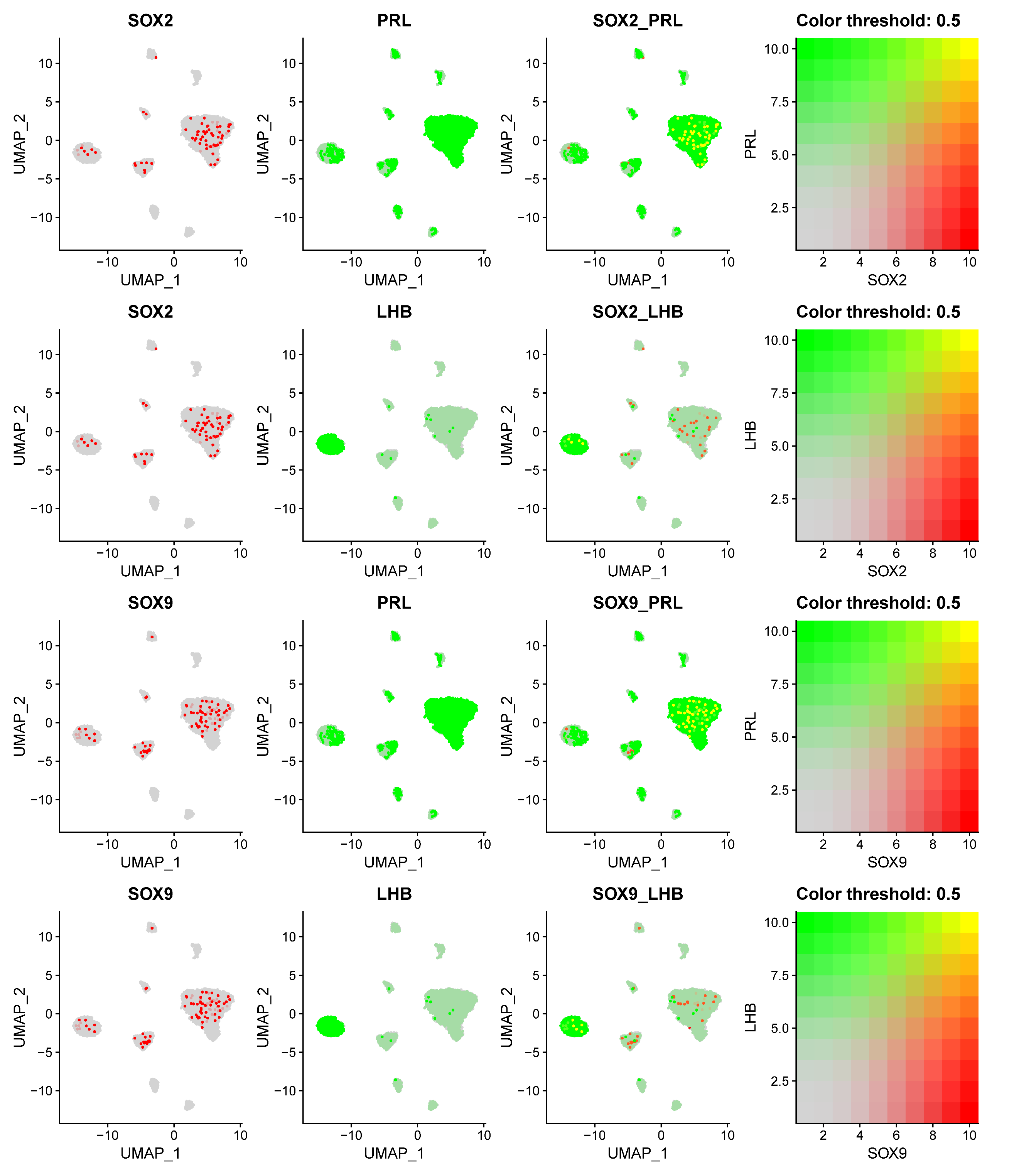


**Figure S12.** Visualization of the co-expression of *SOX2/SOX9* with pituitary *LH/PRL* cells. Gene expression levels are color coded. Red/Green indicates the high expression level and gray indicates the low expression level. Cells abundantly co-expressing *SOX2/SOX9* with *LHB*/*PRL* are shown in yellow.
